# Perillaldehyde Improves Parkinson‐Like Deficits by Targeting G3BP Mediated Stress Granule Assembly in Preclinical Models

**DOI:** 10.1002/advs.202412152

**Published:** 2025-02-14

**Authors:** Minglv Fang, Lingling Luo, Youjia Chen, Ying Liu, Yingxuan Yan, Fei Wang, Yan Zou, Huanhu Zhu, Xiaojun Wu, Zhigang Jin, Cheng Huang, Yu Zhang, Shengjie Fan

**Affiliations:** ^1^ School of Pharmacy Shanghai University of Traditional Chinese Medicine Shanghai 201203 China; ^2^ School of Life Science and Technology ShanghaiTech University Shanghai 201210 China; ^3^ The Affiliated Hospital of Jiangxi University of Traditional Chinese Medicine Nanchang 330006 China; ^4^ College of Life Sciences Zhejiang Normal University Jinhua 321004 China; ^5^ Shanghai‐MOST Key Laboratory of Health and Disease Genomics NHC Key Lab of Reproduction Regulation Shanghai Institute for Biomedical and Pharmaceutical Technologies Shanghai 200237 China

**Keywords:** α‐synuclein, G3BP, histone deacetylase 6, liquid–liquid phase separation, Parkinson's disease, perillaldehyde, stress granules

## Abstract

Stress granules (SGs) fulfill a pivotal role in host defense mechanisms, by sequestering both mRNA and protein via the process of liquid–liquid phase separation (LLPS). In this study, we showed that perillaldehyde (PAE), a natural occurring compound, bound directly to the core protein of SGs, Ras GTPase‐activating protein‐binding protein 1/2 (G3BP1/2), thereby inducing the assembly of SGs through the LLPS of G3BP/RNA complexes in vitro. Moreover, in Parkinson's disease (PD) models using *Caenorhabditis elegans* (*C. elegans*) and mice, PAE administration prompted SG formation, enhanced eIF2α phosphorylation, shielded dopaminergic neurons from toxic insults, mitigated α‐synuclein (α‐syn) aggregation, and improved PD‐like motor disorders. In addition, these findings revealed that the interaction between G3BP1 and histone deacetylase 6 (HDAC6) inhibited the functions of cytoplasmic HDAC6 and reduced α‐syn aggregation in cells and worms. Notably, the inhibition of SG assembly via *gtbp‐1* and *tiar‐1* RNAi effectively counteracted the beneficial effects of PAE in *C. elegans*. Collectively, these results imply that PAE may exert neuroprotective effects by targeting G3BP‐mediated SG formation, thereby safeguarding dopaminergic neurons from toxic damage.

## Graphical abstract

This study uncovers that stress granules (SGs) are involved in the pathogenesis of Parkinson's disease (PD). Perillaldehyde (PAE) can improve Parkinson‐like deficits in preclinical models by directly binding to G3BP and instigating liquid–liquid phase separation (LLPS) of G3BP. These results suggest that targeting G3BP‐mediated SG formation holds promise as a therapeutic strategy for PD.

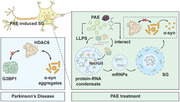

## 1 Introduction

Parkinson's disease (PD) manifests through the aggregation of α‐synuclein (α‐syn), loss of dopaminergic neurons, and neuroinflammation within the substantia nigra, impacting ∼16.1 million individuals worldwide.^[^ ^1^ ^]^ The pathogenesis of PD remains largely unclear, which has hindered the development of the interventions. To date, no therapeutic agent has been identified capable of halting the progress of PD. Stress granules (SGs) are membraneless, reversible cytoplasmic RNA‐protein complexes in eukaryotic cells, closely related to various human diseases.^[^ ^2^ ^]^ These structures are vital for mRNA translation regulation, acting as a cellular defense mechanism during adverse conditions. Beyond mRNA management during stress responses, SGs also serving as signaling hubs by recruiting key signaling pathway components.^[^ ^3^ ^]^ For example, SGs sequestered Raptor to inhibit stress‐induced hyperactivation of mTOR signaling and resultant apoptosis in cancer cells.^[^ ^4^ ^]^ SGs are dynamic entities, assembling swiftly through liquid‐liquid phase separation (LLPS) in response to various stressors such as oxidative stress, viral infection, and endoplasmic reticulum stress.^[^ ^5^ ^]^ Thus, the dynamic shuttling of signaling proteins between SGs and the surrounding cytosol can modulate protein activity and impact related cell physiology during stress. Misfolded proteins or mutations in RNA‐binding proteins (RBPs) can cause abnormal SG formation and toxic protein aggregation. Emerging evidence suggests that defects in SG formation, resulting from disorders in SG dynamics or deficiencies in SG components, may heighten cellular vulnerability to stress, thereby exacerbating neurodegeneration,^[^ ^6^ ^]^ and is implicated in several neurodegenerative diseases, including amyotrophic lateral sclerosis (ALS) and Alzheimer's disease (AD).^[^ ^7^ ^]^

Reversible phosphoryaltion of eukaryotic translation initiation factor 2α (eIF2α) accounts for the transient and reversible nature of SGs.^[^ ^8^ ^]^ Assembly of typical SGs is induced by phosphoryaltion of eIF2α, while dephosphoryaltion of eIF2α induces disassembly of these SGs after stress withdrawal or cellular adaptation to stress. The main downstream effect of eIF2α phosphorylation is the arrest of global translation at the initiation steps via limiting the availability of ternary complex GTP‐eIF2‐Met tRNA. The translationally stalled mRNAs, translation initiation factors and other RNA‐binding proteins are then recruited by Ras‐GTPase‐activating protein (SH3 domain)‐binding protein 1/2 (G3BP1/2) via LLPS to form SGs.

Recent studies have elucidated the pivotal roles of G3BP1/2 as the central hub of core SG network. These proteins function as a molecular switch that initiates LLPS of SG components to assemble SGs.^[^ ^9^ ^]^ G3BP1/2 protein‐protein interaction (PPI) network is essential for stress resilience in stress‐sensitive neurons. Intriguingly, SG proteins are enriched in the G3BP1 interactome in unstressed cells. This pre‐existing G3BP1/2‐PPI network facilitates the rapid assembly of SGs in response to stress.^[^ ^10^ ^]^ However, proteins associated with neurodegenerative diseases, such as poly (GR) and mutant superoxide dismutase 1 (SOD1) in amyotrophic lateral sclerosis, exhibit aberrant interactions with G3BP1/2, leading to the formation of cytoplasmic inclusions that sequester SG proteins and disrupt SG dynamics.^[^ ^11^ ^]^ Similarly, various mutant proteins associated with Charcot‐Marie‐Tooth type 2 (CMT2) also bind aberrantly to G3BP1/2, thereby disrupting the G3BP1/2‐centric SG network and rendering neurons more vulnerable to stress.^[^ ^6a^ ^]^ Therefore, a robust G3BP1‐PPI network confers stress resilience to neurons by maintaining structural and functional integrity of SGs. In addition, the interactions between G3BP1/2 and aggregate‐prone proteins, such as tau protein in AD and ATXN2/3 in spinocerebellar ataxia (SCA), may counteract protein aggregation, highlighting the neuroprotective roles of G3BP1/2‐PPI network.^[^ ^12^ ^]^ It seems that altered G3BP1/2‐PPI is a hallmark feature in the pathogenesis of neurodegenerative diseases, including ALS, AD, CMT2, and SCA. However, it remains unclear whether G3BP1/2‐PPI is also disrupted in PD, and whether such alterations lead to a neurotoxic outcome. In addition, the absence of G3BP1 results in enhanced short‐term potentiation (STP) and long‐term depression in the CA1 area of the hippocampus, and increase intracellular calcium levels and calcium release in response to (S)‐3,5‐Dihydroxyphenylglycine. These findings suggest that G3BP1/2‐dependent SG formation is crucial for neuronal plasticity and calcium homeostasis in neurodegenerative diseases.^[^ ^13^ ^]^ Moreover, G3BP1 is deficient in brain samples from PD patients, and G3BP1 depletion promotes α‐syn aggregation,^[^ ^14^ ^]^ suggesting a protective effect of SGs in PD. Thus, targeting G3BP‐mediated SG formation may potentially shield neurons from adverse stresses in PD.

Histone deacetylase 6 (HDAC6) is a unique cytoplasmic member of histone deacetylase family, known for its dual functionality to deacetylate cytoplasmic nonhistone proteins such as tubulin and heat shock protein 90 (HSP90), as well as to bind to ubiquitinated proteins. The interaction between HDAC6 and G3BP1 pre‐existed in unstressed cells,^[^ ^15^ ^]^ which may facilitate the recruitment of HDAC6 to SGs upon stress. Recent study has revealed that HDAC6 is among the 36 proteins that constitute the core SG network.^[^ ^9a^ ^]^ HDAC6 contributes significantly to the normal dynamics of SGs, including both the assembly and disassembly of SGs. On the one hand, HDAC6 promotes SG formation under various stress by deacetylating another SG core protein, DDX3X, and mediating the motor protein‐driven movement of SG proteins along microtubules, leading to SG maturation featured by the coalescence of small SGs into larger SGs.^[^ ^15^, ^16^ ^]^ On the other hand, HDAC6 facilitates the disassembly of SGs via acetylation of Lysine‐376 of G3BP1.^[^ ^17^ ^]^ Additionally, HDAC6 regulates cellular response pathways to cytotoxic ubiquitinated aggregates and neuroinflammation in certain protein aggregation diseases. In the brains of PD patients, HDAC6 and phospho‐HDAC6 colocalize with pathological protein aggregates, including α‐syn positive Lewy bodies, and directly interact with α‐syn, indicating their involvement in intraneuronal protein homeostasis in parkinsonism.^[^ ^18^ ^]^ Studies in diverse PD animal models have demonstrated that the inhibition of HDAC6 may ameliorate behavioral deficits, suppress the α‐syn‐induced dopamine (DA) neuron loss, regulate α‐syn oligomer levels, and alleviate the nigrostriatal DA neurons injury.^[^ ^19^ ^]^ However, the association of HDAC6‐mediated SG formation and its pathological role in PD remains unclear.

Perillaldehyde (PAE) is a monocyclic terpenoid compound naturally occurring in Perilla leaves that commonly utilized as spices in foods. Recent studies have unveiled a multitude of beneficial effects of PAE, including antioxidant, antifungal, antiviral, anti‐inflammatory, antibacterial, antiatherosclerotic, and antitumor properties.^[^ ^20^ ^]^ Additionally, PAE exerts antidepressive effects by activating brain‐derived neurotrophic factor (BDNF) signaling in the hippocampus of mice.^[^ ^21^ ^]^ Moreover, PAE has been found to inhibit neuronal damage, improve cognitive capabilities,^[^ ^22^ ^]^ and attenuate cerebral ischemia‐reperfusion injury in the brain^[^ ^23^ ^]^ and spinal cord in rats.^[^ ^24^ ^]^ However, it remains unknown whether PAE possesses anti‐Parkinsonian properties. In the present study, we demonstrate that PAE directly targets G3BP to induce the formation of SGs that sequester cytoplasmic HDAC6. Furthermore, we observe anti‐Parkinsonian effects of PAE in *Caenorhabditis elegans* (*C. elegans*) and mouse models, thus providing a novel therapeutic strategy for PD.

## 2 Results

### 2.1 PAE Induces the Assembly of SGs In Vitro

**Figure 1:**
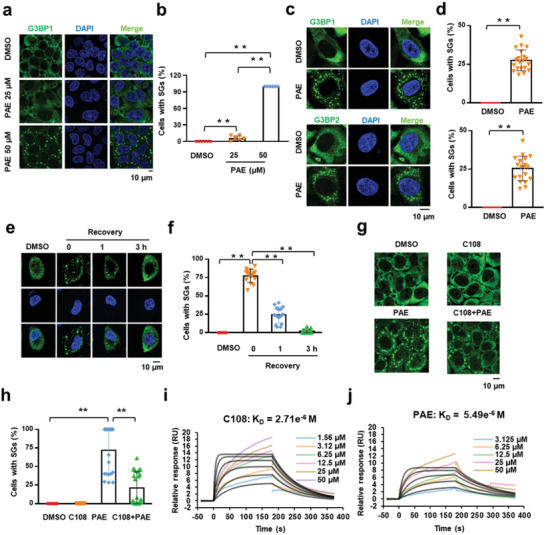
PAE promotes SG assembly by targeting G3BP1. a) The effect of PAE on SGs is concentration‐dependent. HeLa cells were treated with the indicated PAE concentrations for 1 h, followed by immunostaining for G3BP1 (green). b) Statistical analysis of the SG‐assembly index shown in panel (a). c) 16HBE cells were treated with PAE 200 × 10^−6^ m for 1 h, followed by immunostaining for G3BP1 and G3BP2 (green). d) Statistical analysis of the SG‐assembly index shown in panel (c). e) The dynamics of SG disassembly in PAE‐treated cultures. HeLa cells were pretreated with PAE (1 × 10^−6^ m) for 1 h and fixed at the indicated time‐points during the recovery and stained with G3BP1 antibody. Representative images for each time point are shown. f) Statistical analysis of the SG‐disassembly index shown in panel (e). g) HeLa cells were exposed to C108 (10 × 10^−6^ m, 24 h) or PAE (1 × 10^−3^ m,1 h), or were pretreated with 10 × 10^−6^ m C108 for 24 h prior to PAE 1 × 10^−3^ m for 1 h, followed by immunostaining for G3BP1 (green). h) Statistical analysis of the SG‐assembly index shown in panel (g). i,j) Surface plasmon resonance analysis of C108 and PAE interaction with G3BP1 protein. Analytes at different concentrations in duplicate (Compound 108 [C108]: 1.56, 3.12, 6.25, 12.5, 25, 50 × 10^−6^ m and [PAE]: 3.125, 6.25, 12.5, 25, 50 × 10^−6^ m) were injected over the biosensor surface with high levels of immobilized G3BP1 protein. The data fit to a 1:1 binding model is represented by a black line. The concentrations of soluble analytes and affinity constant (KD) values are indicated in the inset to the figures. Data are shown as the mean ± SEM, *p < 0.05, **p < 0.01.

To screen the compounds that could activate SG formation, we developed a cell‐based screen system using HeLa cell line stably expressing EGFP‐G3BP2 fusion protein (EGFP‐G3BP2/HeLa).^[^ ^2^ ^]^ From an 800‐natural compound library, PAE was shown to promote SG formation with high efficiency. Consistently, PAE promoted SG assembly in a dose‐dependent manner by immunofluorescence against endogenous G3BP1 in HeLa cells (**Figure** **1**a,b). Similarly, a significant increase in SG assembly was observed following PAE treatment in HeLa cells transfected with both EGFP‐G3BP1 and EGFP‐G3BP2 (Figure 1c,d), suggesting that PAE can induce SG formation. Since SG disassembly is linked to translational recovery, we then assessed the dynamics of SG disassembly in HeLa cells pretreated with PAE for 1 h, and recovered for the indicated time‐points. Approximately 3 h recovery compared with control group, SGs were completely disassembly and cell morphology remained normal (Figure 1e,f). To investigate whether stress preconditioning affects the induction of SG by PAE, sodium arsenite (SA)‐pretreated EGFP‐G3BP2/HeLa cells were used to observed PAE‐induced SG. The results showed that PAE induced SG assembly after recovery from SA stress (Figure S1a,b, Supporting Information). Similarly, SA also induced SG formation in PAE‐pretreated cells (Figure S1a,b, Supporting Information). These results indicate that PAE induced‐SG is dynamic and reversible. We then investigated whether PAE could induce SG formation in neuroblastoma SH‐SY5Y cells. By immunostaining of G3BP1 and TIA‐1, we observed colocalization of G3BP1 with TIA‐1 in SH‐SY5Y cells after exposure to PAE (Figure S1c,d, Supporting Information), which is similar to that after exposure to SA. Upon the withdrawal of PAE, PAE‐induced SGs would completely disassemble within 1 h, suggesting that PAE‐induced SGs are also reversible in SH‐SY5Y cells (Figure S1c,d, Supporting Information). Taken together, PAE may promote SG assembly in multiple cell lines.

### 2.2 PAE Directly Targets G3BP1 and Enhances Phase Separation of G3BP1 in vitro

G3BP1/2 are SG core proteins and crucial for SG formation. To test whether the suppression of G3BP could block the SG promoting effects of PAE, we used G3BP1/2 inhibitor C108 together with PAE in SG inducing experiments.^[^ ^25^ ^]^ We found that the SG formation was markedly inhibited by C108 in cells treated with PAE (Figure 1g,h). To investigate whether PAE directly targets G3BP1 protein, we performed surface plasmon resonance (SPR) measurements in vitro. Compared to compound C108, PAE was showed similar binding ability with equilibrium dissociation constant (*K*_d_) values of 2.71 and 5.49 × 10^−6^ m, respectively (Figure 1i,j). Taken together, these results support that PAE might directly target G3BP to assemble SGs.

**Figure 2:**
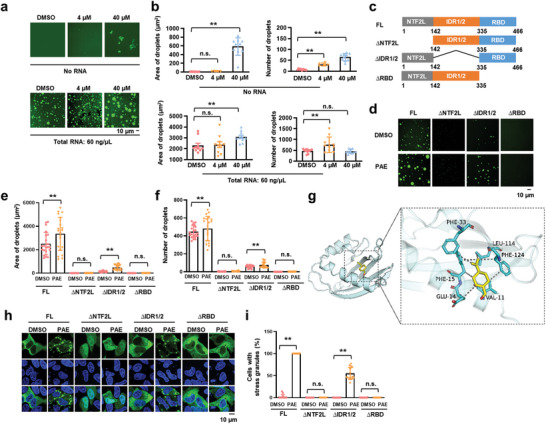
PAE enhances phase separation of G3BP1 in vitro. a) LLPS of G3BP1‐EGFP protein with DMSO or PAE, with or without the addition of 60 ng µL^−1^ total RNA (from HeLa cell). The concentration of protein was 15 × 10^−6^ m. b) Column charts display the droplets size and number in panel (a). c) Schematic domain structures of the G3BP1 protein used in the study. d) LLPS of individual domain constructs of G3BP1 protein with 100 ng µL^−1^ total RNA in the absence or presence of PAE. e,f) Column charts display the droplets size and number in panel (d). g) The interaction model of PAE with NTF2L domain of G3BP1. h) G3BP1/2 dKO HeLa cells were transfected with indicated G3BP1 constructs, exposed to PAE (1 × 10^−3^ m; 1 h), and stained for G3BP1. i) Statistical analysis of the SG‐assembly index shown in panel (h). Data are shown as the mean ± SEM, *p < 0.05, ** *p* < 0.01, n.s., not significant.

Recently several studies have shown that SG assemble through RNA‐dependent LLPS of G3BP.^[^ ^9^ ^]^ Thus, we investigated whether PAE can affect G3BP1 protein phase separation in vitro. We purified mEGFP‐tagged recombinant G3BP1 protein and incubated it with PAE. At physiological salt concentration (150 × 10^−3^ m NaCl), it triggered the formation of flocculent condensates (**Figure** **2**a,b). Furthermore, addition of total RNA triggered the robust LLPS of G3BP1, and PAE increased the number and size of droplets in a concentration‐dependent manner compared with DMSO control (Figure 2a,b), indicating that PAE could target and promote G3BP1 phase separation in vitro.

G3BP1 and G3BP2 proteins share similar structures, an N‐terminal NTF2‐like (NTF2L) domain, a central disordered region 1/2 (IDR1/2) and a C‐terminal RNA‐binding domain (RBD). The NTF2L domain of G3BP1/2 mediates dimerization function and is critical for RNA‐independent assembly. To dissect the contribution of the various G3BP1 domains to LLPS, we generated constructs in which NTF2L, IDR1/2, and RBD were deleted (Figure 2c). We found that the truncation of NTF2L and RBD domains inhibited the LLPS in the presence of RNA, while IDR1/2 is dispensable for LLPS (Figure 2d), suggesting that NTF2L and RBD domains are required for G3BP1 phase separation. Furthermore, we incubated PAE and G3BP1 truncations with RNA. Upon mixing, PAE significantly increased G3BP1 droplet size and number, depending on the existence of NTF2L and RBD (Figure 2e,f). It also excluded the possibility that PAE functions as a molecular crowding agent like polyethylene glycol (PEG) to promote LLPS of G3BP1. To analyze PAE binding site in G3BP1, we carried out molecular docking assay, which showed that PAE closely contacted PHE‐33, PHE‐124, VAL‐11, PHE‐15, GLU‐14, LEU‐114 residues on the NTF2L domain by hydrophobicity (Figure 2g), suggesting that PAE may interact with the NTF2L domain of G3BP1. Next, we assessed the ability of G3BP1 truncations to rescue PAE‐induced SG assembly in G3BP1/2 double knockout (G3BP1/2 dKO) cells. Consistent with in vitro LLPS behavior, PAE did not promote SG formation with the deletion of NTF2L or RBD of G3BP1, whereas the deletion of IDR1/2 did restore SG assembly by PAE treatment (Figure 2h,i). Therefore, PAE promotes SG assembly via LLPS of G3BP1, a process that requires NTF2L and RBD domain of G3BP1.

### 2.3 PAE Stabilizes G3BP1 Condensates by LLPS and Activates PKR/eIF2α Signaling

**Figure 3:**
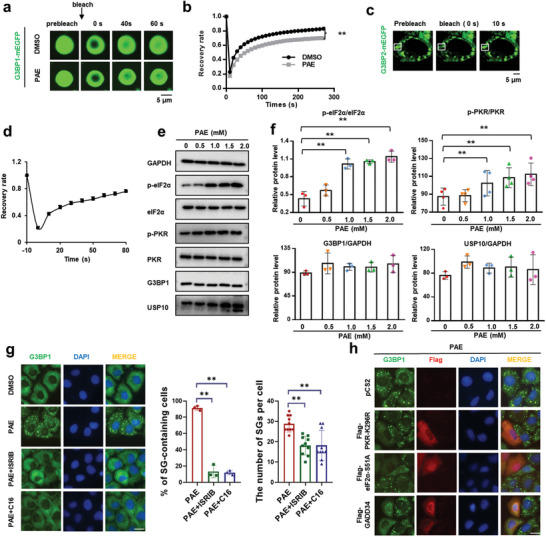
PAE stabilizes G3BP1 condensates by LLPS. a) FRAP of G3BP1‐RNA liquid droplets. Bleaching was performed at the indicated time points. Time 0 indicates the start of recovery after photobleaching. b) Plot showing the time course of recovery after photobleaching G3BP1‐RNA droplets. Data are shown as the mean ± SD (*n* > 10). c) HeLa cells transfected with G3BP2‐mEGFP were treated with 1 × 10^−3^ m PAE for 1 h to induce SGs, and FRAP of SGs. d) Plot showing the time course of recovery after photobleaching SG droplets. e) PAE activates the PKR pathway and increases p‐eIF2α protein. The expression of related proteins in HeLa cells was detected by WB after the treatment of PAE at different concentrations. f) Corresponding to panel (e) statistical graph of gray values of protein bands using imageJ. g) HeLa cells were treated with ISRIB or C16 for 24 h after reached a density of 50–60% on coverslips. In the presence of inhibitors, cells were then treated with PAE for 1 h. Endogenous G3BP1 was labeled with rabbit anti‐G3BP1 (green). h) HeLa cells were transfected with pCS2 as a control vector, Flag‐GADD34, Flag‐eIF2α‐S51A, or Flag‐PKR‐K296R, and treated with PAE (100) × 10^−6^ m for 1 h. Endogenous G3BP1 was labeled with rabbit anti‐G3BP1 (green), and the Flag tag was labeled with mouse anti‐Flag (red). Note that SGs were successfully induced by PAE in non‐transfected cells (Flag negative). Scale bars: 10 µm. Data are shown as the mean ± SEM (*n* = 3), *p < 0.05, **p < 0.01.

To further characterize the liquid nature of G3BP condensates by PAE, we analyzed the fluorescence recovery after photobleaching (FRAP) of LLPS droplets. We observed a rapid recovery of the fluorescence in G3BP1 droplets, which was consistent with previous studies (**Figure** **3**a,b). By contrast, PAE mixing in G3BP1 droplets, we observed a slower recovery of the fluorescence, indicating that G3BP1 condensates are more stable (Figure 3a,b). In addition, we expressed G3BP2‐mEGFP and analyzed SG dynamics or properties in PAE‐treated cells by fluorescence recovery after photobleaching (FRAP) assay. The results showed that SGs were typical liquid‐like as previously observed (Figure 3c,d). Taken together, the data indicate that G3BP condensates could be hardened by small molecule PAE, which may be facilitated SG assembly through G3BP phase separation.

Mechanistically, SG formation is mainly induced by phosphorylation of eIF2α at the serine residue 51 or interfered with the function of translation initiation factor 4F complex (consisting of eIF4E, eIF4A, and eIF4G). Four types of stress‐sensing serine/threonine kinases, including heme‐regulated inhibitor (HRI), general control nonderepressible 2 (GCN2), protein kinase R (PKR), and PKR‐like ER kinase (PERK) are responsible for phosphorylation of eIF2α. We therefore assessed the effect of various concentrations PAE on the levels of phosphorylated eIF2α (p‐eIF2α) in cells. It is worth noting that the level of p‐eIF2α was significantly elevated in a dose‐dependent manner by PAE (Figure 3e,f). Further, a small but statistically significant increase in the level of PKR phosphorylation (p‐PKR) was observed after increased PAE concentrations. To confirm that PAE induced SGs via PKR‐eIF2α phosphorylation cascade, cells were pretreated with PKR inhibitor C16 or ISRIB that could reverse the effect of p‐eIF2α and treated with PAE.^[^ ^26^ ^]^ As shown in Figure 3g, pretreatment of C16 or ISRIB resulted in a significant decrease in the percentage of SG‐containing cells and the number of SGs per cell compared to PAE group. Additionally, the ability of PAE to induce SG formation was abolished in cells transfected with PKR‐K296R, a dominant negative form of PKR that disrupts PKR phosphorylation.^[^ ^26a^ ^]^ Antagonization of eIF2α phosphorylation by overexpression of eIF2α nonphosphorylated mutant eIF2α‐S51A or GADD34, the regulatory subunit of protein phosphatase 1 (PP1) that dephosphorylates p‐eIF2α,^[^ ^27^ ^]^ also blocked PAE‐induced SG formation (Figure 3h). Taken together, we conclude that PAE induces SGs primarily through PKR‐mediated phosphorylation of eIF2α.

### 2.4 PAE Induces SG Formation and Increases the Resistance to Stress in *C. elegans*

**Figure 4:**
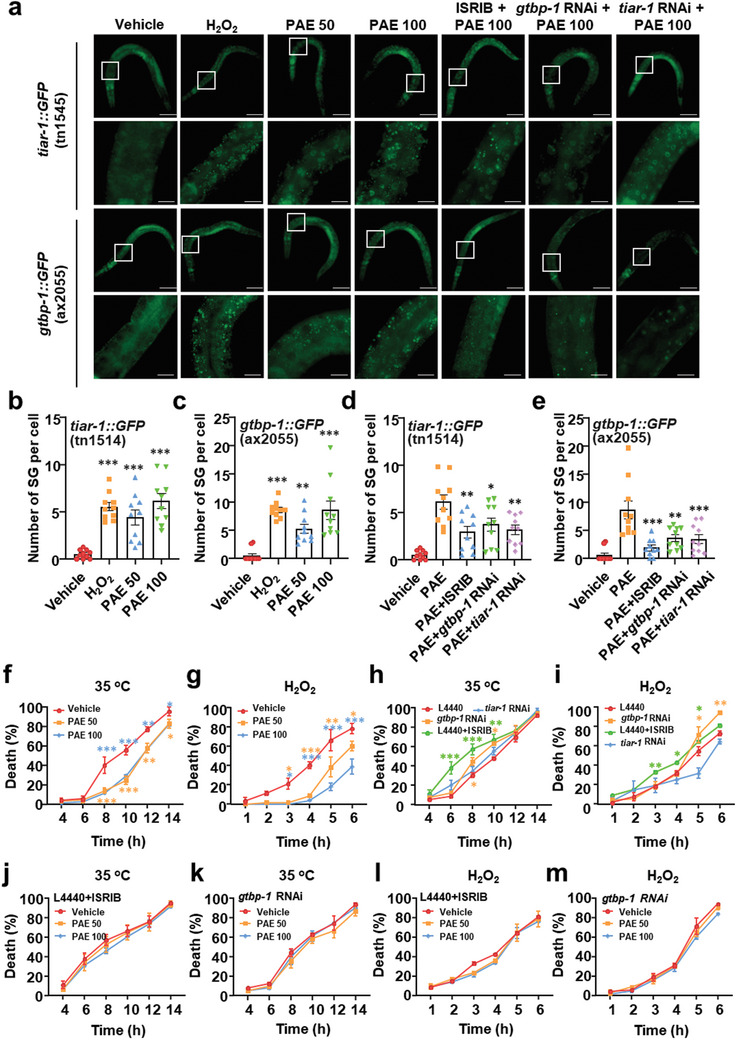
PAE induces SG formation and increases the resistance to stresses in *C. elegans*. a) Transgenic *tiar‐1::GFP* and *gtbp‐1::GFP* worms were feeding with *tiar‐1* or *gtbp‐1* RNAi bacteria or treated with ISRIB from L1 to L3 stage, SG formation was captured after the worms were exposed to hydrogen peroxide (2 × 10^−3^ m) or treated with PAE for 1 h. Scale bars: 50 µm, 10 µm (enlarged image). b–e) Quantification of the experiment performed in panel (a). Each dot represents average number of SGs in 10–15 cells/worm, *n* = 10. ***p < 0.001 versus vehicle, **p < 0.01 versus vehicle in (b) and (c). ***p < 0.001 versus PAE, **p < 0.01 versus PAE, *p < 0.05 versus PAE in (d) and (e). f) Percentage of death on ≈20 N2 worms treated or untreated with PAE from L4 stage to adult day 5 under acute heat stress (35 °C, 14 h) conditions, *n* = 3. The following experimental conditions for heat stress are the same. ***p < 0.001 versus vehicle, **p < 0.01 versus vehicle, *p < 0.05 versus vehicle. g) Percentage of death on ≈30 N2 worms treated or untreated with PAE from L4 stage to adult day 5 under acute oxidative stress induced by hydrogen peroxide (2 × 10^−3^ m, 6 h) conditions, *n* = 2. The following experimental conditions for oxidative stress are the same. ***P < 0.001 versus vehicle, **p < 0.01 versus vehicle, *p < 0.05 versus vehicle. h,i) Percentage of death on *tiar‐1* RNAi, *gtbp‐1* RNAi, or ISRIB‐treated *C elegans* under heat stress (h) or oxidative stress (i). Data are shown as the mean ± SEM. ***p < 0.001 versus L4440, **p < 0.01 versus L4440, *p < 0.05 versus L4440. j–m) *tiar‐1* RNAi, *gtbp‐1* RNAi or ISRIB abolished PAE effect on stress resistance in worms.

SG assembly and disassembly are multistage, dynamic, and reversible processes, which make them difficult to track in vivo.^[^ ^5^, ^28^ ^]^ *C. elegans* are transparent and easy to observe their microscopic structures under microscope. Thus, we used transgenic *C. elegans* that expressing *gtbp‐1*::GFP and *tiar‐1*::GFP (homolog of G3BP1/2 and TIA‐1 in *C. elegans*, respectively) to monitor SG dynamics according to a previous report.^[^ ^29^ ^]^ As shown in **Figure** **4**a–c, tiar‐1 and gtbp‐1 granules were accumulated in worms after hydrogen peroxide (H_2_O_2_)‐induced oxidative stress. These foci also appeared in worms treated with PAE in a dose‐dependent manner, suggesting that PAE induces SGs in *C. elegans*. Next, we used *tiar‐1* and *gtbp‐1* RNAi and ISRIB to suppress SG formation and treated with PAE in the worms (Figure 4a,d,e). PAE‐induced SGs were markedly diminished in ISRIB, *tiar‐1* and *gtbp‐1* RNAi treated worms, suggesting that PAE induces SG formation depending on SG core proteins tiar‐1 and gtbp‐1 as well as the phosphorylation of eIF2α. Moreover, PAE‐induced SGs in worms were disassembled completely after cellular adaptation to PAE within 3 h (Figure S1e,f, Supporting Information), suggesting that PAE‐induced SGs are also dynamic and reversible in worms.

It is well known that SGs can promote cell survival via their stress resistance property.^[^ ^30^ ^]^ Previous studies have also suggested that SG assembly may promote worm survival and extend the lifespan by enhancing organismal stress resistance.^[^ ^31^ ^]^ Thus, we assayed the resistance to various stresses of PAE treated worms. The results showed that PAE significantly diminished mortality of worms treated by acute heat stress and oxidative stress (Figure 4f,g). ISRIB or *gtbp‐1* RNAi have been shown to accelerate cell or worm death under unfavorable conditions.^[^ ^32^ ^]^ Under conditions of heat stress at 35 °C or H_2_O_2_‐induced oxidative stress, ISRIB, *tiar‐1* and *gtbp‐1* RNAi increased worm death (Figure 4h,i). While, in PAE treated worms, the stress resistance induced by PAE was eliminated by ISRIB and *gtbp‐1* RNAi (Figure 4j–m). These results suggest that PAE is able to promote cell survival under unfavorable circumstances, which depends on SG formation.

### 2.5 PAE Exerts Neuroprotective Effect on *C. elegans* Models of PD via SG Induction

**Figure 5:**
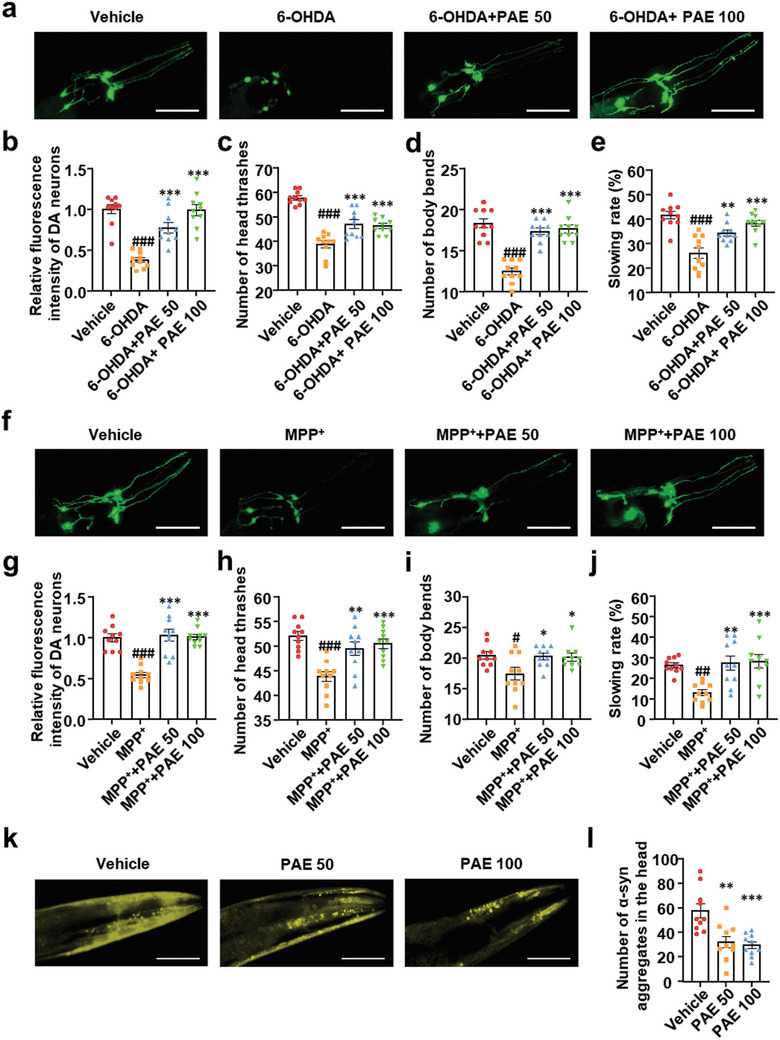
PAE has neuroprotective effect on PD *C. elegans*. a) Transgenic UA57 (dat‐1p::GFP, dat‐1p::CAT‐2) worms were exposed to 6‐OHDA (5 × 10^−3^ m) for 2 h at L3 stage and treated or untreated with PAE in the whole process. Dopaminergic neurons in head (four CEPs and two ADEs) of worms were captured after 72 h treatment. b) Quantification of the experiment performed in panel (a). Column charts display relative fluorescence intensity of dopaminergic neurons (fold of vehicle) measured by ImageJ software, *n* = 10. ***p < 0.001 versus 6‐OHDA, ###*p* < 0.001 versus vehicle. c–e) Behavioral phenotyping of worms was observed after 72 h treatment of 6‐OHDA. Column charts respectively display, from left to right, number of head thrashes/20 s in M9 buffer, number of body bends/20 s in a NGM plate without food, slowing rate/20 s, n = 10. Slowing rate = (*N*_Body bends without food_ – *N*_Body bends with food_)/*N*_Body bends without food_. ***p < 0.001 versus 6‐OHDA, **p < 0.01 versus 6‐OHDA, ###*p* < 0.001 versus vehicle. f,g) Fluorescence intensity of dopaminergic neurons in UA57 when exposed into MPP^+^ (2 × 10^−3^ m) for 2 h at L3 stage, *n* = 10. ***p < 0.001 versus 6‐OHDA, ###*p* < 0.001 versus vehicle. h–j) Behavioral phenotyping of worms was observed after 72 h treatment of MPP^+^. Column charts respectively display, from left to right, number of head thrashes/20 s in M9 buffer, number of body bends/20 s in a NGM plate without food, slowing rate/20 s, *n* = 10. ***p < 0.001 versus MPP^+^, **p < 0.01 versus MPP^+^, *p < 0.05 versus MPP^+^, ###*p* < 0.001 versus vehicle, ##*p* < 0.01 versus vehicle, #*p* < 0.05 versus vehicle. k) Transgenic NL5901 (unc‐54p::α‐synuclein::YFP) worms were treated or untreated with PAE from L1 to Day5. l) Quantification of the experiment performed in (k). Column charts display the number of α‐syn aggregates in the head of worms, *n* = 10. **p < 0.01 versus vehicle, *p < 0.05 versus vehicle. Data are shown as the mean ± SEM. Scale bars: 100 µm.

Emerging evidence has shown that aging‐related neurodegenerative diseases are closely related to SGs. For example, senescent individuals or patients with neurodegeneration fail to maintain SG assembly.^[^ ^33^ ^]^ Defects in SG dynamics would sensitize neuronal cells to various stresses thus inducing neurodegeneration.^[^ ^6^ ^]^ Therefore, we investigated whether PAE, the SG‐inducer, exhibits pharmacological effects on PD firstly. The *cat‐2* (homolog of TH in *C. elegans*) transgenic strain UA57 (dat‐1p::GFP, dat‐1p::CAT‐2), which marks visible dopaminergic neurons with GFP, was exposed of the synchronous L3 larvae to 6‐OHDA or MPP^+^, the toxic metabolite of the neurotoxin 1‐methyl‐4‐phenyl‐1, 2, 3, 6‐tetrahydropyridine (MPTP) for 72 h. Compared with vehicle group, these two classic neurotoxins diminished the relative fluorescence intensity of dopaminergic neurons, body bends frequency, head thrashing frequency, basal slowing rate in UA57 worms significantly (**Figure** **5**a–j). PAE treatment significantly reduced damage to dopaminergic neurons and improved dopamine‐dependent behavioral deficits in the PD worms (Figure 5a–j). Moreover, we utilized NL5901(unc‐54p::alphasynuclein::YFP) transgenic strain, expressing wild‐type human α‐syn fused to yellow fluorescent, to observe whether PAE could inhibit α‐syn aggregation. Worms were treated with/without PAE from L1 to day 5. Figure 5k,l showed that PAE reduced the number of α‐syn aggregates in the head of worms significantly. In short, these results indicate that PAE may improve the PD like impairment in *C. elegans*.

Next, we further examined whether the neuroprotective effect of PAE depends on SG assembly. We used ISRIB, *tiar‐1* and *gtbp‐1* RNAi to inhibit SG assembly, and treated the worms with PAE. The results showed that ISRIB, *tiar‐1* and *gtbp‐1* RNAi suppressed SG assembly, but did not impact dopaminergic neurons in worms (Figure S1g,h, Supporting Information). However, in SG suppressed worms, PAE did not reverse dopaminergic neurons damage and dopamine‐dependent behavior impairment including body bends frequency, head thrashing frequency, basal slowing rate caused by 6‐OHDA (Figure S2a–o, Supporting Information). We then assayed how PAE reverses the inhibitory effects of MPP^+^ on SG formation. As shown in Figure S3a–o (Supporting Information), ISRIB treatment, *tiar‐1* and *gtbp‐1* RNAi inhibited the protective effect of PAE on dopaminergic neurons in MPP^+^ treated worms, suggesting that PAE may play a dopaminergic neuroprotective role in worms treated with MPP^+^ through inducing SG assembly.

### 2.6 PAE Exerts Neuroprotective Effects on PD Mice via Enhancing SG Formation in the Striatum and the SNpc

**Figure 6:**
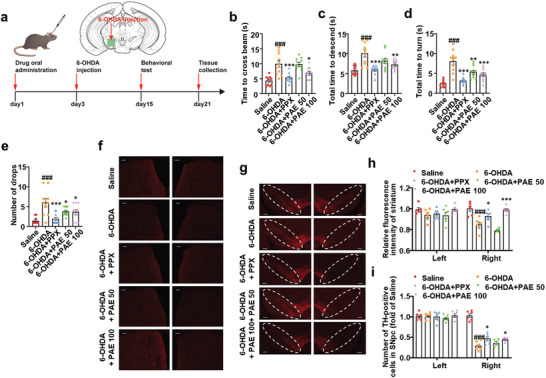
PAE has neuroprotective effect on 6‐OHDA‐induced mice models of PD. a) Timeline of drug treatment, modeling, behavioral test and tissue collection. b) Balance beam test, *n* = 10. c,d) Pole test, *n* = 10. e) Rotarod test, *n* = 10. f) TH‐positive neurofilament staining via immunofluorescence in striatum region of brain slice. The right images are the 6‐OHDA injection side. Scale bar: 100 µm. g) TH‐positive cells staining via immunofluorescence in SNpc region of brain slice. Scale bar: 200 µm. The right images are the 6‐OHDA injection side. h) Quantification of the experiment performed in (f). Each dot represents relative fluorescence intensity of TH‐positive neurofilament in a striatum slice (fold of saline), *n* = 6. i) Quantification of the experiment performed in panel (g). Each dot represents relative number of TH‐positive cells in a SNpc slice (fold of saline), *n* = 6. Data are shown as the mean ± SEM. ***p < 0.001 versus 6‐OHDA, **p < 0.01 versus 6‐OHDA, *p < 0.05 versus 6‐OHDA, ###*p* < 0.001 versus Saline.

To confirm the effect of PAE on PD, 6‐OHDA and MPTP‐induced PD mice were analyzed. In 6‐OHDA‐induced PD model, mice were unilaterally injected 6‐OHDA into SNpc via stereotaxic injection apparatus as the timeline shown in **Figure** **6**a. Mice showing constant unilateral rotation were considered successfully modeling. Behavioral tests including the beam test, the pole test, and the rotarod test were examined for motor coordination of mice. The results showed that 6‐OHDA‐induced PD mice spent longer time to cross the beam, to descend or to turn round in the pole, and were more vulnerable to fall from the rotarod, indicating 6‐OHDA induced behavioral impairment in mice (Figure 6b–e). Mice treated with PAE (100 mg kg^−1^) spent a shorter time to cross the beam, to descend or to turn round in the pole, and exhibited stronger grasping capability and motor coordination in the rotarod, suggesting that PAE may alleviate 6‐OHDA‐induced Parkinson‐like behavioral impairment similar to positive drug pramipexole (PPX), though lower dosage of PAE (50 mg kg^−1^) did not significantly shorten crossing time of the beam and descending time of the pole (Figure 6b–e). In the brain sections, TH positive cells, a maker of dopaminergic neurons, in unilateral SNpc (right) and TH‐positive neurofilaments in unilateral striatum (right) were reduced by 6‐OHDA compared with those in saline treated controls (left), suggesting that PAE could reverse 6‐OHDA‐induced dopaminergic neurodegeneration (Figure 6f–i).

**Figure 7:**
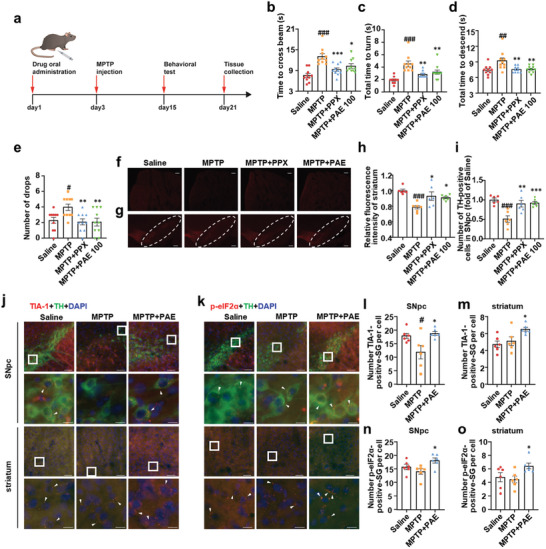
PAE has neuroprotective effect on MPTP‐induced mice models of PD. a) Timeline of drug treatment, modeling, behavioral test, and tissue collection. b) Balance beam test, *n* = 10. c,d) Pole test, *n* = 10. e) Rotarod test, *n* = 10. f) TH‐positive neurofilament staining via immunofluorescence in striatum region of brain slice. The right images are the 6‐OHDA injection side. Scale bar: 100 µm. g) TH‐positive cells staining via immunofluorescence in SNpc region of brain slice. Scale bar: 200 µm. The right images are the 6‐OHDA injection side. h) Quantification of the experiment performed in panel (f). Each dot represents relative fluorescence intensity of TH‐positive neurofilament in a striatum slice (fold of saline), *n* = 6. i) Quantification of the experiment performed in panel (g). Each dot represents relative number of TH‐positive cells in a SNpc slice (fold of saline), *n* = 6. j) Colocalization study of TIA‐1 and TH via immunofluorescence in striatum and SNpc. The white arrow points to the TIA‐1‐positive SGs. Scale bars: 50 µm, 10 µm (enlarged image). k) Colocalization study of p‐eIF2α and TH via immunofluorescence in striatum and SNpc. The white arrow points to the p‐eIF2α granules. Scale bars: 50 µm, 10 µm (enlarged image). l) A partial quantification of experiment (SNpc) performed in panel (j). Each dot represents average number of TIA‐1‐positive SGs in ≈10 TH‐positive cells of SNpc slice, *n* = 6. m) A partial quantification of experiment (striatum) performed in panel (j). Each dot represents average number of TIA‐1‐positive SGs in ≈10 cells of striatum slice, *n* = 6. n) A partial quantification of experiment (SNpc) performed in panel (k). Each dot represents average number of p‐eIF2α granules in ≈10 TH‐positive cells of SNpc slice, *n* = 6. o) A partial quantification of experiment (striatum) performed in panel (k). Each dot represents average number of p‐eIF2α granules in ≈10 cells of striatum slice, *n* = 6. Data are shown as the mean ± SEM. ***p < 0.001 versus 6‐OHDA, **p < 0.01 versus 6‐OHDA, *p < 0.05 versus 6‐OHDA, ###*p* < 0.001 versus Saline, ##*p* < 0.01 versus Saline, #*p* < 0.05 versus Saline.

In MPTP‐induced PD mice, MPTP was given intraperitoneally for 5 consecutive days as showing in the timeline (**Figure** **7**a). After the injection of MPTP on the 5th day, the modeled mice exhibited trembling and slow moving. Results from the behavioral tests showed that MPTP treatment prolonged the crossing time of the beam, descending the time and turning time of the pole, and increased frequency of drops from the rotarod, showing MPTP induced behavioral impairment in mice. Similar to PPX, PAE treatment significantly improved MPTP‐induced PD like behavioral impairment in mice (Figure 7b–e). Neuropathological examination showed that MPTP also induced TH‐positive cell loss in the SNpc and TH‐positive neurofilament damage in the striatum of the mice. PAE treatments rescued TH‐positive cell loss in the SNpc and TH‐positive neurofilament damage in the striatum (Figure 7f–i). Together, PAE may relieve toxin induced PD‐like behavior disorders and alleviate dopaminergic neuronal degeneration in mice.

The disruption of SG dynamics and consequent stress vulnerability in neurodegenerative diseases has been well elaborated.^[^ ^11^ ^]^ Indeed, a variety of classic neurotoxins causing dopaminergic neurons damage, such as paraquat, rotenone, and MPP^+^, have been demonstrated to affect SG dynamics, suggesting that SGs may be associated with PD.^[^ ^34^ ^]^ Next, we asked whether SGs are involved in the dopaminergic neuron protective effects of PAE. Colocalization study of TIA‐1 and TH via immunofluorescence was carried out to determine whether PAE facilitated SG formation in the striatum and the SNpc. MPTP treatment showed that there were less number of TIA‐1‐positive granules in the SNpc (Figure 7j,l), but no difference in the number of TIA‐1‐positive SGs in the striatum as well as the number of p‐eIF2α granules in the striatum and the SNpc (Figure 7j–o). PAE treatment increased the number of TIA‐1‐positive granules in TH‐positive region of the striatum and the SNpc of MPTP treated mice when compared with vehicle controls (Figure 7j–m). Moreover, p‐eIF2α levels were also increased by PAE compared with the controls (Figure 7k–o), which consistent with the findings in vitro (Figure 3e,f). Taken together, our results suggest that PAE may increase SG formation in TH‐positive region of the striatum and the SNpc via enhancement of p‐eIF2α signaling.

### 2.7 Interaction between G3BP1 and HDAC6 is Disturbed in PD but Restored by PAE

**Figure 8:**
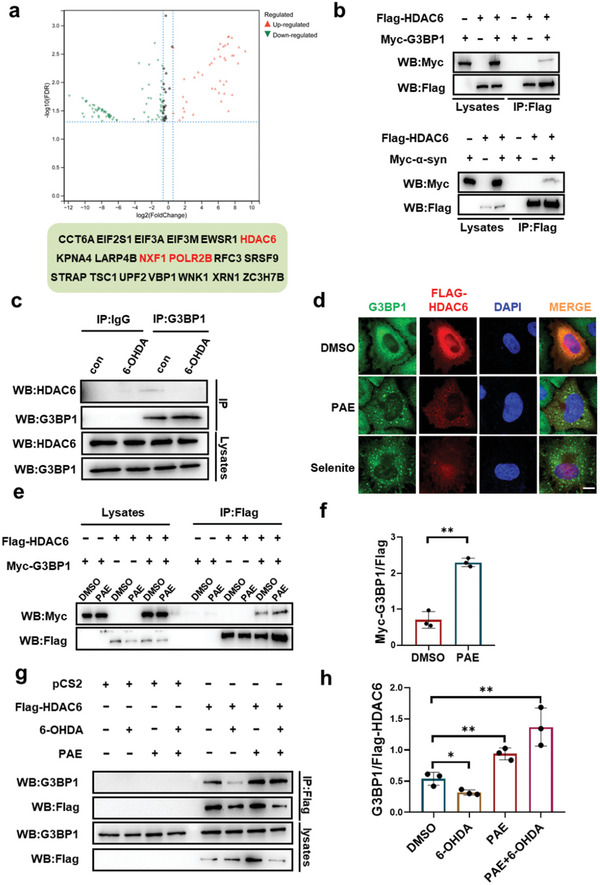
PAE recruits HDAC6 to SGs and potentiates G3BP1‐HDAC6 interaction. a) Volcano plots showing differential G3BP1 PPI in 6‐OHDA‐treated versus untreated SH‐SY5Y cells identified by TurboID and MS. Student's *t* test with the correction to multiple hypotheses by FDR adjusted *p* < 0.05. A list of proteins with decreased binding to G3BP1 was shown with SG core proteins in red. b) (Top) HEK293T cells transfected with pCS2‐Flag‐HDAC6, pCS2‐Myc‐G3BP1, or both were subjected to immunoprecipitation with an anti‐Flag antibody. The presence of G3BP1 in the immunoprecipitates was assessed by western blotting with an anti‐Myc antibody. (Bottom) HEK293T cells transfected with pCS2‐Flag‐HDAC6, pCS2‐Myc‐α‐syn, or both were subjected to immunoprecipitation with an anti‐Flag antibody. The presence of α‐syn in the immunoprecipitates was assessed by western blotting with an anti‐Myc antibody. c) SH‐SY5Y cells were treated with 100 × 10^−6^ m 6‐OHDA for 24 h. The supernatant was then subjected to immunoprecipitation with an anti‐G3BP1 antibody. The presence of HDAC6 in the immunoprecipitates was assessed by western blotting with an anti‐HDAC6 antibody. d) HeLa cells transfected with pCS2‐Flag‐HDAC6 were treated with 100 × 10^−6^ m PAE for 1 h or 1 × 10^−3^ m selenite for 2 h followed by immunostaining for G3BP1 (green) and Flag (red). Scale bars: 10 µm. e) HEK293T cells transfected with pCS2‐Myc‐G3BP1, pCS2‐Flag‐HDAC6, or both were treated with DMSO or 100 × 10^−6^ m PAE for 1 h and subjected to immunoprecipitation with an anti‐Flag antibody. The presence of G3BP1 in the immunoprecipitates was assessed by western blotting with an anti‐Myc antibody. f) Statistical analysis of co‐IP shown in panel (e), *n* = 3. g) SH‐SY5Y cells transfected with mock vector or pCS2‐Flag‐HDAC6 were pre‐treated with DMSO or 100 × 10^−6^ m PAE for 1 h, then treated with DMSO or 100 × 10^−6^ m 6‐OHDA for 24 h and subjected to immunoprecipitation with an anti‐Flag antibody. The presence of G3BP1 in the immunoprecipitates was assessed by western blotting with an anti‐G3BP1 antibody. h) Statistical analysis of co‐IP shown in panel (g), *n* = 3. Data are shown as the mean ± SEM, *p < 0.05, **p < 0.01.

PAE executes a neuroprotective role dependent on G3BP1 in PD pathogenesis, which encourages us to investigate the underlying mechanism of G3BP1 in PD. Next, we employed G3BP1‐TurboID‐based proximity labeling with mass spectrometry (MS) in 6‐OHDA‐treated SH‐SY5Y cells, a cell‐based model commonly employed to mimic PD pathology, to reveal the interacting proteins of G3BP1. We identified 124 differential hits out of 2908 biotinylated proteins by comparison of the G3BP1 interactome after 6‐OHDA treatment (**Figure** **8**a). Among these, 43 proteins exhibited an increase in the interaction with G3BP1, while only 4 proteins (9.30%) were reported SG components.^[^ ^10^ ^]^ In contrast, 81 proteins exhibited a decrease in the interaction with G3BP1 after 6‐OHDA treatment, and 19 proteins (23.46%) were reported SG components.^[^ ^9a^ ^]^ Thus, the pre‐existing G3BP1 PPI network was disturbed in 6‐OHDA‐treated SH‐SY5Y cells. Compared to the untreated cells, 6‐OHDA‐treated SH‐SY5Y cells were defective in stress‐induced assembly of SGs and cell survival under stress conditions (Figure S4, Supporting Information). We found that α‐syn did not interact with G3BP1 (Figure S5a,b, Supporting Information), suggesting that G3BP1 may not directly modulate α‐syn aggregation. Of the 19 SG proteins with decreased G3BP1 binding ability, we particularly focused on HDAC6, which is an SG protein and also contributes to dopaminergic neurotoxicity in diverse PD animal models.^[^ ^19^ ^]^ Next, we confirmed that HDAC6 was in complex with G3BP1 and α‐syn respectively and that the interaction between G3BP1 and HDAC6 was attenuated by 6‐OHDA treatment in SH‐SY5Y cells (Figure 8b,c), indicating a neuroprotective role of G3BP1 and HDAC6 interaction. Interestingly, HDAC6 was recruited to PAE‐induced SGs but not selenite‐induced SGs (Figure 8d). We also found that HDAC6 was presented in a subset of SGs (Figure S5c,d, Supporting Information). Recruitment of HDAC6 to PAE‐induced SGs might result from the enhanced the interaction between HDAC6 and G3BP1 by PAE (Figure 8e,f). In addition, pretreatment of PAE restored the interaction of HDAC6 and G3BP1 attenuated by 6‐OHDA (Figure 8g,h), as well as the ability of SG assembly impaired by 6‐OHDA (Figure S4, Supporting Information). Furthermore, the enhancing effects of PAE on the interaction between HDAC6 and G3BP1 was the most prominent among a panel of SG inducers (Figure S5e, Supporting Information). Given that SGs serve as a signaling hub to rewire cell signaling by sequestering signaling proteins,^[^ ^3a^ ^]^ our results suggest that PAE can prevent HDAC6‐meidated neurotoxicity by restoring the interaction between G3BP1 and HDAC6 to sequester HDAC6 within SGs.

### 2.8 Deficiency of hda‐6 Attenuates the Neuroprotective Effect of PAE in *C. elegans* Models and In Vitro

We investigated the involvement of PAE‐induced SG in the clearance of α‐syn in *C. elegans*. ISRIB, *tiar‐1* and *gtbp‐1* RNAi were used to inhibit SG assembly, and treated the worms with PAE. In SG suppressed worms, PAE failed to inhibit α‐syn aggregation (Figure S6a–e, Supporting Information), suggesting that PAE‐induced SG is involved in the process of α‐syn aggregation inhibition. Then, we further examined whether hda‐6 (homolog of HDAC6 in *C. elegans*) is associated with α‐syn aggregation. We firstly examined the effect of HDAC agonist ITSA‐1 on NL5901. As shown in Figure S6f,g (Supporting Information), ITSA‐1 treatment barely affected α‐syn aggregation in worms while PAE treatment inhibited α‐syn aggregation in worms regardless of the presence or absence of ITSA‐1. Subsequently, we used selective HDAC6 inhibitor Tubastatin A (TBS) and *hda‐6* (*F41H10.6*) RNAi to suppress the expression of *hda‐6* in worms. As shown in Figure S6h–k (Supporting Information), *hda‐6* inhibition significantly diminished the number of α‐syn aggregates in worms, while PAE treatment did not further improve the inhibitory effect on α‐syn aggregation in hda‐6 suppressed worms.

**Figure 9:**
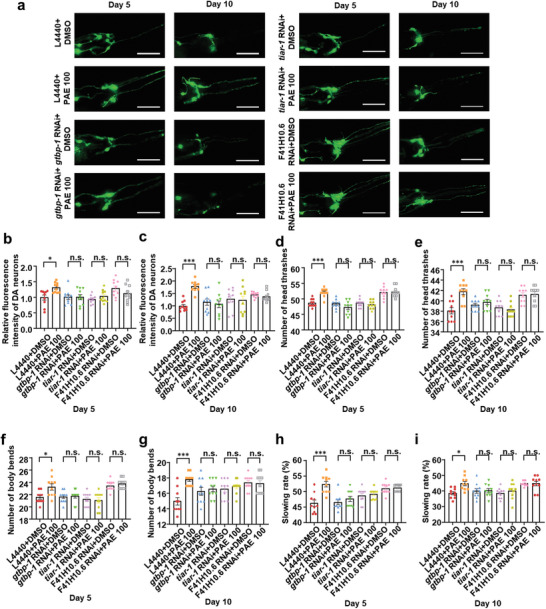
Deficiency of *hda‐6* attenuates the neuroprotective effect of PAE in α‐syn aggregation *C. elegans*. a) Transgenic UA44 worms were treated or untreated with *gtbp‐1* RNAi, *tiar‐1* RNAi and *F41H10.6* (*hda‐6*) RNAi and PAE until adult Day 5 and Day 10. b,c) Quantification of the experiment performed in panel (a). Column charts display relative fluorescence intensity of dopaminergic neurons (fold of vehicle) measured by ImageJ software. d,e) Number of head thrashes/20 s in M9 buffer. f,g) Number of body bends/20 s in a NGM plate without food. h,i) Slowing rate/20 s. Data are shown as the mean ± SEM, *n* = 10, ***p < 0.001 versus vehicle, **p < 0.01 versus vehicle, *p < 0.05 versus vehicle, n.s., not significant. Scale bars: 100 µm.

To further evaluate the alleviating effects of PAE on neurotoxicity induced by α‐syn, dopaminergic neurons expressed α‐syn transgenic strains UA44 (dat‐1p::α‐synuclein, dat‐1p::GFP) was used. As shown in **Figures** **9**a–i and S7a–i (Supporting Information), PAE treatment ameliorated dopaminergic neuron damage and behavioral deficiency caused by α‐syn both in the 5th and 10th day of adulthood in worms, suggesting that PAE treatment can improve α‐syn‐induced neurotoxicity. Next, ISRIB treatment, *tiar‐1* and *gtbp‐1* RNAi was performed to inhibit SG assembly. In SG suppressed worms, PAE failed to alleviate α‐syn‐induced neurotoxicity (Figure 9a–i; Figure S7a–i, Supporting Information). Also, PAE was unable to exhibit the neuroprotective effect on dopaminergic neurons expressed α‐syn worms treated with *hda‐6* RNAi and TBS (Figure 9a–i; Figure S7a–i, Supporting Information). Furthermore, either PAE treatment or HDAC6 knockdown alleviated 6‐OHDA‐induced neurotoxicity in SH‐SY5Y cells. However, HDAC6 knockdown fails to synergize with PAE for neuroprotection, supporting the neuroprotective effects of PAE depend on HDAC6 (Figure S8, Supporting Information). Taken together, these results suggest that PAE may alleviate α‐syn‐induced neurotoxicity in worms via SG or hda‐6 mediated α‐syn aggregation suppressing in *C. elegans*.

## 3 Discussion

As our results showed, PAE could induce SG formation both in vitro and in vivo. It is well‐known that SGs are membraneless organelles formed in eukaryotic cells to protect against oxidative stress, apoptosis, inflammation, and viral infection under acute stress conditions.^[^ ^30^ ^]^ Moreover, PAE induced SG formation through G3BPs/gtbp‐1 and TIA/tiar‐1 signaling, while their absence or inhibition largely attenuated SGs both in vitro and in *C. elegans*. We also demonstrated that PAE treatment could promote worm survival under acute heat stress and oxidative stress, which may depend on gtbp‐1 and tiar‐1. gtbp‐1 and tiar‐1 are crucial components of SGs in worms. It has been reported that *gtbp‐1* RNAi was detrimental to survival of worms when exposed to heat stress.^[^ ^29^, ^32^ ^]^ The SG inhibitor ISRIB can increase toxic‐induced cell death via reverse of eIF2α phosphorylation, suggesting that insufficient SGs may reduce cellular resistance to stress.^[^ ^32a^ ^]^ Our results showed that the inhibition of SGs by both *gtbp‐1* RNAi and ISRIB treatment accelerated worm death under acute heat stress and oxidative stress. Additionally, the inhibition of SGs eliminated the effects of PAE on enhancing stress resistance in worms, indicating that the survival‐promoting effects of PAE depend on SG formation. Stress resistance privileges the organism against damage from stressors, and the capacity of PAE to enhance stress resistance implies that PAE may promote neuronal survival under stressful conditions.

G3BP1/2 are core components of SGs. Upon stress, G3BP1/2 undergo conformational changes through the phosphorylation of its IRDs, which promotes the dimerization of the NTF2L domain. The dimerized NTF2L, in turn, promotes the exposure of the RBD, thereby enhancing G3BP1‐RNA interactions and maintaining LLPS and SG assembly.^[^ ^9a^ ^]^ Previously, we showed that SARS‐CoV‐2 nucleocapsid protein interacts with G3BP to inhibit LLPS of G3BP, and disrupted SG assembly, highlighting the importance of G3BP1/2 in inhibiting virus production.^[^ ^35^ ^]^ Recently, it has been reported that G3BP2 may directly interact with Tau, mask its microtubule‐binding region and inhibiting Tau aggregation. In human brains and brain organoids, Tau pathology is significantly elevated upon loss of G3BP2.^[^ ^12b^ ^]^ In the present study, we found that PAE may directly bind with G3BP to induce the formation of SGs. First, PAE induced the LLPS of G3BP in vitro, while the G3BP protein lacking of NTF2L, IDR1/2, and RBD regions exhibited no or reduced LLPS, ruling out false interaction between PAE and G3BP proteins or possibility that PAE functions as a molecular crowding agent like PEG to promote LLPS of G3BP1. Second, SPR experiment revealed that PAE directly interacted with G3BP1 protein with similar a binding ability similar to that of G3BP ligand compound C108. Third, molecular docking assay showed that PAE may bind to NTF2L domain of G3BP1. Finally, in *C. elegans*, the loss of *gtbp‐1* attenuated the effects of PAE. Thus, our data support that PAE can directly bind with G3BP1/2 proteins.

Furthermore, we found PAE increased the phosphorylation of eFI2α and PKR. The phosphorylation of eIF2α is the major pathway accounting for SG formation.^[^ ^7a^ ^]^ SGs are induced mostly via the phosphorylation of eIF2α that leads to disruption of translation initiation, and disassembled via reversible dephosphorylation of eIF2α. PKR is an eIF2α kinase, which is able to phosphorylate eFI2α. Therefore, PAE may increase the phosphorylation of PKR, which consequently induced the phosphorylation of eFI2α and the assembly of SGs. Given that chronic stress‐caused insufficient p‐eIF2α levels can impair the protective effect of SGs in neurodegenerative diseases,^[^ ^33a^ ^]^ we speculate that PAE may maintain p‐eIF2α levels to promote SG assembly in neurons with SG assembly defect.

Impressively, SG formation induced by PAE and SG disassembly after PAE withdrawal are typical behaviors of SGs upon a variety of stress, such as sodium arsenite. SGs are dynamic structure that is usually formed within a few minutes after stress treatment, and disassembled within 1–2 h after stress withdrawal. SGs are transient and reversible. However, under pathological states such as ALS disease SGs become persistent, stable and irreversible. We noticed that PAE induced SGs were disassembled after withdrawal, which could reinduce by other SG inducer. Thus, PAE induced SGs may be beneficial to the neurodegeneration progress.

The impact of SGs on PD remains unknown. Recent evidence showed that G3BP1 can inhibit the ubiquitinated α‐syn protein aggregates, suggesting that G3BP1 executes a neuroprotective role in PD by counteracting α‐syn aggregates.^[^ ^36^ ^]^ In the present study, PAE significantly alleviated the PD‐like behavioral disorders and protected against dopaminergic neuron damage induced by MPP^+^/MPTP and 6‐OHDA both in *C. elegans* and mice. Meanwhile, PAE induced SGs in neuroblastoma SH‐SY5Y cells, worms and mouse brains. The protective effect of PAE on dopaminergic neurons vanished when SG assembly was inhibited. Previously, disruptions in SG dynamics have been associated with pathological aggregation in the brain tissue of patients with neurodegenerative diseases.^[^ ^7a^ ^]^ SG assembly disorders may induce cells more sensitive to various stress, exacerbating neurodegeneration.^[^ ^33^ ^]^ Thus, the dopaminergic neuroprotective effects of PAE may depend on SG assembly.

The aggregation of α‐syn in neurons is a central mechanism of the pathogenesis of PD. Although the process of α‐syn aggregation depends on LLPS, there is no evidence that α‐syn and SGs colocalize, indicating that α‐syn is not a component of SGs.^[^ ^37^ ^]^ PAE also displayed the ability to prevent α‐syn aggregating in cells and worms. Our findings, however, showed that PAE failed to decrease the number of α‐syn aggregates when SGs were suppressed. Therefore, SGs induced by PAE may be associated with α‐syn aggregation through indirect interaction. G3BP1 is considered to be neuroprotective in several neurodegenerative diseases. In PD, G3BP1 deficit has been discovered in the brain tissues of PD patients, playing an important role in α‐syn ubiquitination, aggregation, oligomerization and neurotoxicity.^[^ ^36^, ^38^ ^]^ However, G3BP1 does not interact with α‐syn directly, implying that targeting G3BP cannot attenuate α‐syn aggregation. The process of α‐syn aggregation may be influenced by a specific SG component.

**Figure 10:**
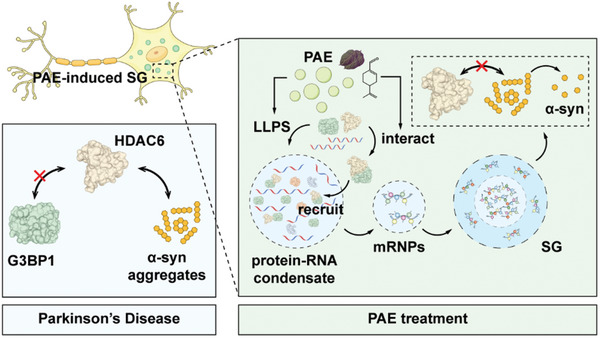
PAE‐induced SG inhibits α‐syn aggregation by recruiting G3BP1‐HDAC6 complexes. In PD, the interaction of G3BP1 with HDAC6 is weakened while HDAC6 accelerates α‐syn aggregation. PAE treatment facilitates LLPS‐driven condensate formation by targeting G3BP1 and in turn assembling SGs. Moreover, the interaction of G3BP1 with HDAC6 is potentiated after PAE treatment prompting SGs to recruit additional HDAC6, which causes a decrease in intracellular HDAC6 and an inhibition of α‐syn aggregation.

By analyzing the differences of G3BP1 PPI between 6‐OHDA‐treated and untreated SH‐SY5Y cells, we focused on HDAC6, a component of SGs that can influence SG dynamics by regulating the acetylation of Lysine‐376 of G3BP1 and motor‐protein‐driven movement.^[^ ^15^, ^17^ ^]^ Some reports showed that HDAC6 can alleviate α‐syn aggregation,^[^ ^39^ ^]^ while other studies showed that HDAC6 deficiency exhibited moderate alterations in behavior and PD pathology in mice. Upon MPTP treatment, motor injury of HDAC6^−/−^ mice was slightly alleviated, while dopamine depletion in the striatum, the number of DA neurons in the substantia nigra, the reduction in DA neuronal terminals, activation of glial cells and α‐syn aggregation were attenuated.^[^ ^40^ ^]^ Similarly, HDAC6 inhibitor tubastatin A can decrease 6‐OHDA‐induced expression of NLRP3, caspase‐1 and IL‐1β, alleviate glial proliferation and dopaminergic neuronal degeneration, reduce oxidative stress, and inhibit α‐syn phosphorylated at serine position 129, indicating that pharmacological inhibition of HDAC6 may protect against dopaminergic neuron damage. HDAC6 is thus considered a potential target for PD treatment.^[^ ^19^, ^41^ ^]^ The complex and controversial role of HDAC6 in PD might be caused by different models and different methods for HDAC6 modulation used in these studies. In the present study, we showed that inhibition of *hda‐6* with RNAi or inhibitors in *C. elegans* significantly relieved α‐syn aggregation, dopaminergic neuron damage and motor defects. Mechanistically, the interaction between G3BP1 and HDAC6 was impaired in PD, leading to defect in SG formation and vulnerability of dopaminergic neurons to stress. However, PAE restored G3BP1‐HDAC6 interaction and SG formation. Thus, our results support that PAE‐induced SGs can serve as a platform for G3BP1‐HDAC6 interaction. On the one hand, PAE‐induced SGs sequester cytoplasmic HDAC6, thereby inhibiting its pathogenic role in PD. On the other hand, the recruitment of HDAC6 to PAE‐induced SGs also allows SG‐localized HDAC6 to maintain SG dynamics and confer stress resilience to dopaminergic neurons (**Figure** **10**).

In summary, our study demonstrated that PAE could bind to G3BP1/2 facilitating SG assembly through LLPS of G3BP/RNA complexes. PAE prompted SG formation, protected dopaminergic neuron from the toxic insults, mitigated α‐syn aggregation, and attenuated PD‐like motor defects in both *C. elegans* and mice. Notably, the inhibition of SG assembly abolished the beneficial effects of PAE in *C. elegans*. Moreover, we showed that G3BP1 interacted with HDAC6 and α‐syn to suppress α‐syn aggregation, and the neuroprotective effects of PAE depended on the interaction between G3BP1 and HDAC6. Collectively, these findings suggest that targeting G3BP‐mediated SG formation could represent a promising therapeutic strategy for PD.

## 4 Experimental Section

### Plasmid Construction

DNA fragments encoding G3BP1, HDAC6, and α‐synuclein were amplified by PCR using Q5 high‐fidelity DNA polymerase (New England Biolabs, Ipswich, MA) and 293T cDNA as template, and cloned into pCS2‐Myc or pCS2‐Flag vector. The coding region for G3BP1 protein deletions (∆NTF2L, ∆IDR1/2, and ∆RBD) were amplified by PCR from a plasmid containing full‐length G3BP1. Exnase (Vazyme, C214‐02‐AF, Nanjing, Jiangsu, China) was used to insert these sequences into the CMV‐Flag, CMV‐GFP, or CMV‐mCherry backbone. All constructs were confirmed by sequencing.

### Cell Culture and Transfection

HeLa, H1‐HeLa, HEK293T, and SH‐SY5Y cells were obtained from ATCC. Human bronchial epithelial cells (16HBE) were purchased from Sigma. Cells were cultured in DMEM (Gibco, C11995500BT, Waltham, MA, USA) supplemented with 10% fetal bovine serum (Gemini, 900 108, Woodland, CA, USA) and 1% penicillin/streptomycin (Gibco, 15 140 122, Waltham, MA, USA). For plasmid transfection, cells were transfected using Lipofectamin 3000 reagent (Invitrogen, L3000008). After transfection for 6 h, the cells were moved to fresh DMEM and cultivated for 48 h.

### Generation of G3BP1/2 Double Knockout Cell Lines

G3BP1/2 double knockout cell lines were generated using small guide RNA (sgRNA) for G3BP1 and G3BP2.^[^ ^42^ ^]^ HeLa cells were transfected with 1 µg of sgRNA and 2 µg of spCas9‐mcherry plasmid using Lipofectamine 3000 transfection kit (Invitrogen, USA). After transfection for 6 h, the cells were moved to fresh DMEM and cultivated for 48 h. Both GFP and mcherry positive cells were harvested from fluorescence‐activated cell sorting (FACS) and targeting sequences was verified by sequencing. Furthermore, the efficiency of G3BP1/2 protein knockdown was verified by Western blot analysis. The sequences of sgRNA used are G3BP1‐sg: aacgtttgtccttgctcctg; G3BP2‐sg: cgcatcaataccaagggtgt.

### Western Blotting

Western blotting was performed as previously described.^[^ ^35^ ^]^ Briefly, cells were lysed in RIPA buffer and the extracts were resolved by sodium dodecyl sulfate polyacrylamide gel electrophoresis (SDS‐PAGE) and transferred to polyvinylidene fluoride (PVDF) membranes (Millipore, IPVH00010, Darmstadt, Germany). The membranes were blocked and incubated with primary antibodies at 4 °C overnight. The protein bands were detected with horseradish peroxidase‐conjugated secondary antibodies and Immobilon Western enhanced chemiluminescent solution (Millipore, WBKLS0100, Darmstadt, Germany). The protein levels were normalized by probing the same blots with GAPDH antibody. The following antibodies were used: Flag (Sigma‐Aldrich, #F1804 and #F7425), Myc (Sigma‐Aldrich, #M5546), G3BP1 (Proteintech, #13057‐2‐AP), G3BP2 (Proteintech, #16276‐1‐AP), HDAC6 (Proteintech, #12834‐1‐AP and Santa Cruz Biotech, #28 386).

### RT‐qPCR

The total RNA of H1‐HeLa cells was purified using TRIzol (TaKaRa, 9108). 1 µg RNA was reverse transcribed into cDNA using Reverse Transcriptase (Vazyme, R223‐01‐AB). Gene expression was assayed by real‐time PCR using 2 × ChamQ SYBR (Vazyme, Q331‐AA) on ABI ViiA 7 real‐time PCR system (Applied Biosystems, Carlsbad, USA). The mRNA levels of were normalized using GAPDH as an internal control. Measurements were performed in triplicate for each biological sample. Viral genomic RNA expression was normalized to that of the DMSO‐treated control. Then, qPCR primers targeting GAPDH (AGAAGGCTGGGGCTCATTTG and AGGGGCCATCCACAGTCTTC), HRV16 viral genome (CCTCACATCCCTTAGCCACA and ACCACGTGTGTCCCTAACAT) were used.

### Co‐Immunoprecipitation

HEK293T, SH‐SY5Y, and HeLa cells transfected were collected and lysed with RIPA buffer contained with protease inhibitor cocktail (Thermo Fisher scientific, 78 443). The cell lysates were incubated on ice for 15 min and centrifuged at 16 000 g at 4 °C for 20 min, the supernatant was incubated with pre‐equilibrated anti‐Flag beads (Bimake, B26101) overnight at 4 °C. Then, the beads were washed with PBS supplemented with 0.05% V/ Tween‐20 to remove nonspecific binding proteins. 1×loading buffer containing SDS were added into each sample and heated at 98 °C for 10 min to elute bound proteins. The supernatant was harvested and used for SDS‐PAGE analysis.

### Liquid–Liquid Phase Separation

Protein expression and purification was performed as previously described.^[^ ^35^ ^]^ Briefly, protein‐coding fragments were synthesized by Genscript, and deletions were generated by PCR. Then sequences were cloned into pET‐28a vector and confirmed by sequencing. Proteins were expressed in *E. coli* BL21 (DE3) (Trans, CD601). Proteins were eluted from the polypropylene column (QIAGEN, 34 964, Hilden, Germany) and were further purified by size exclusion with a Superdex‐200 column on an AEKTA purifier (GE Healthcare Life Sciences, Boston, USA). The fractions were analyzed by SDS‐PAGE, concentrated and stored at −80 °C. LLPS experiments were performed at room temperature and conducted in 150 mmol L^−1^ NaCl, pH 8.0. LLPS of G3BP1 was induced by addition of indicated concentrations of PAE or total RNA. Total RNA was isolated from HeLa cells using TRIzol (TaKaRa). The samples were mixed in low binding tubes (Axygen) and transferred in 96‐well glass bottom plate (Cellvis) and observed under a Nikon spinning disk microscope equipped with a 60×oil immersion objective. All imaged were captured within 5 min after LLPS induction.

### Surface Plasmon Resonance (SPR) Assay

Experiments were performed on a Biacore 8K using CM5 sensor chips. A cell on the CM5 sensor chip was activated with a mixture of 200 × 10^−6^ m 1‐ethyl‐3‐(3‐dimethylaminopropyl) carbodiimide (EDC) and 50 × 10^−6^ m *N*‐hydroxysuccinimide (NHS) at 10 µL min^−1^ for 10 min. EGFP‐tagged G3BP1 protein was diluted to 10 µg mL^−1^ with 10 × 10^−3^ m sodium acetate solution at pH 4.0 and protein was then immobilized on the surface of the cell at 10 µL min^−1^ for 5 min for two repetitive runs. The cell was then blocked with 1 m ethanolamine (10 µL min^−1^ for 10 min). Compound 108 and PAE was diluted to a series of concentrations (all in PBST containing 5% DMSO) and was flowed at 30 µL min^−1^, contact time 120 s and dissociation time 60 s in each run. Data were collected using Biacore 8K Control software (GE Healthcare) and were subtracted by DMSO control. Association and dissociation constants were obtained by global fitting of the data to a 1:1 Langmuir binding model using Biacore Insight Evaluation software (GE Healthcare).

### FRAP Analysis

The experiment was performed using a Nikon spinning disk microscope equipped with two laser systems. A region of the indicated protein droplets was bleached by a 488/561 nm wavelength laser with a light intensity of 80%. Only the center of the droplets was bleached. Fluorescence intensity recovery data were recorded at a given time interval. Fluorescence intensity was obtained using FIJI (National Institutes of Health, Bethesda, MD, USA). Fluorescence intensities of regions of interest (ROIs) were subtracted by background intensity and then normalized by prebleached intensities of the ROIs. The FRAP recovery curve was fit to the formula described previously.

### Molecular Docking Assay

The crystal structure of G3BP1 protein used for docking was obtained from the PDB database (PDB ID: 4FCJ), and the 3D structure of PAE was obtained from the PUBCHEM database. AutoDock Vina 1.2.3 software^[^ ^43^ ^]^ was used to process the receptor protein, including the removal of water molecules, salt ions, and small molecules. Subsequently, the docking box was set up so that it wrapped around the entire protein structure. In addition, PAE and G3BP1 were converted to the PDBQT format by ADFRsuite 1.0.^[^ ^44^ ^]^ For docking, the exhaustiveness of the global search was set to 32 and the rest of the parameters were left at their default settings. The output docked conformations with the highest scores were considered to be the binding conformations and finally the docking results were visualized and analyzed using PyMol 2.5.5.^[^ ^45^ ^]^

### Biotin Proximity Labeling Assay (TurboID)

TurboID assay was performed to map protein‐protein interactions in cells as previously described.^[^ ^46^ ^]^ SH‐SY5Y cells were transfected with pCS2‐hG3BP1‐TurboID‐GFP plasmids for 36–48 h, and then treated with biotin (Sangon Biotech, Shanghai, China) for 15 min. Cells were washed with PBS, lysed in RIPA buffer containing protease inhibitors at 4 °C for 15 min, and centrifuged for 5 min (14 000 rpm, 4 °C). The supernatant was transferred to a new sterile tube, and centrifuged twice. 25 µL supernatant was used as input. Streptavidin beads (Smart Lifesciences, Changzhou, China) was added to the supernatant, and the mixture was incubated overnight at 4 °C. The next day, after washing with RIPA buffer, one tenth of the bead suspension were boiled in SDS loading buffer for 5 min, and the supernatants were used for western blotting. The sterile tube containing remaining beads were transported on dry ice to APExBIO Biotech (Shanghai, China) for mass spectrometry analysis.

### Caenorhabditis Elegans Strains and Maintenance

The following *C. elegans* strains were used in this study: Wild‐type N2 Bristol, DG3922 (tn1545[tiar‐1::GFP::tev::s]), JH3199 (ax2055 [gtbp‐1::GFP]), UA57 (bals4[dat‐1p::GFP, dat‐1p::CAT‐2]), NL5901 (pkls2386[unc‐54p::alphasynuclein::YFP]). These strains were obtained by CGC (Caenorhabditis Genetics Center). UA44 (baIn11[dat‐1p::α‐synuclein, dat‐1p::GFP]) was obtained from Prof. Xiajing Tong of ShanghaiTech University. The worms were cultured on nematode growth medium (NGM) plates seeded with *Escherichia coli* OP50 as food at 20 °C, according to standard protocols.^[^ ^47^ ^]^

### RNA Interference Experiments

L4440 [empty vector (EV)] and RNAi bacterial *gtbp‐1*, *tiar‐1*, and *hda‐6* were obtained from Prof. Huanhu Zhu. They were grown overnight at 37 °C in LB plus 100 µg mL^−1^ ampicillin (Solarbio, A8180, China), and then seeded onto NGM plates containing 1 × 10^−3^ m IPTG (Shandong Sparkjade Biotechnology Co., Ltd., SJ‐MB0016, China) and 100 µg mL^−1^ ampicillin. Worms were cultured on the RNAi NGM plates from L1 larvae stage throughout the experiment.

### tiar‐1 and gtbp‐1 Granule Quantification

The age‐synchronized L3 worms carrying *tiar‐1*::GFP  or and *gtbp‐1*::GFP were washed in M9 buffer, and exposed to M9 buffer solution which adds concentrated bacterial solution and respectively contains 1% DMSO/2 × 10^−6^ m H_2_O_2_/50 × 10^−6^ m PAE/100 × 10^−6^ m PAE for 1 h under dark condition. Then the worms were fixed with anhydrous ethanol and visualized with a fluorescence microscope. tiar‐1::GFP  or and gtbp‐1::GFP foci was quantified by ImageJ according to reference.^[^ ^29^ ^]^

### Stress Resistance Assays

Heat stress and oxidative stress tests were conducted as previous study.^[^ ^48^ ^]^ The age‐synchronized L4 worms were treated with PAE for 5 d. Then worms were exposed to 35 °C for 14 h or 2 × 10^−6^ m H_2_O_2_ for 6 h, the number of dead worms were recorded every 2 h.

### Dopaminergic Neurodegeneration Assays

The dopaminergic neurons degeneration of transgenic strains UA57 was induced by neurotoxin 6‐OHDA or MPP^+^ as described.^[^ ^49^ ^]^ The age‐synchronized L1 larvae were preconditioned with PAE to the L3 stage. These worms were exposed to 6‐OHDA (10 × 10^−3^ m, Macklin, China) or MPP^+^ (2 × 10^−6^ m, Sigma, Germany) solution for 2 h under dark circumstance. Then, worms were retransferred onto NGM plates with PAE and treated for 72 h. After treatment, worms were anesthetized with 100 × 10^−3^ m sodium azide, and dopaminergic neurons were visualized with a fluorescence microscope. In addition, α‐synuclein‐induced dopaminergic neurons degeneration were observed in transgenic strains UA44 which is cultivated until 5th and 10th day of adulthood.

### Behavioral Test of *C. elegans*

Food‐sensing behavior assays and thrashing assays were conducted as described.^[^ ^49^ ^]^ For food‐sensing test, 6‐OHDA or MPP^+^ treated worms were picked to the center of NGM plates with or without OP50 lawn and allowed to acclimatize for 60 s. Then, the number of body bending of worms were recorded in 20 s. Each change in direction of movement was considered a body bend. Meanwhile, the slowing rate was calculated as follows: slowing rate = (*N*_without OP50_ − *N*_with OP50_)/*N*_without OP50_, as previously describe.^[^ ^50^ ^]^ Similarly, body bends and slowing rate in dopaminergic neurons expressed α‐syn worms were measured by the above method.

For thrashing assays, the 6‐OHDA or MPP^+^ treated worms were placed in a 10 µL drop of M9 buffer on a glass slide and allowed to acclimatize for 20 s. Then, the number of head thrashing of worms were recorded in 20 s. Each single thrash was considered a head thrash. Similarly, head thrashes in dopaminergic neurons expressed α‐syn worms were measured by the above method.

### α‐Synuclein Aggregation Quantification

Transgenic strains NL5901 were used to evaluate α‐syn accumulation.^[^ ^49^ ^]^ The age‐synchronized L1 larvae were preconditioned with PAE for 5 d. Then, worms were anesthetized with 100 × 10^−3^ m sodium azide, and α‐synuclein aggregates in body wall muscle cells were visualized with a fluorescence microscope.

### Mice and Treatments

Eight‐week‐old male C57BL/6 mice were obtained from Shanghai Laboratory Animal Center of Chinese Academy of Science (Shanghai, China). All mice were housed under a 12 h light/dark cycle at controlled temperature (22 ± 1 °C) conditions with ad libitum access to food and water, and allowed to acclimate for two weeks. All procedures were approved by the Experimental Animal Ethical Committee at Shanghai University of Traditional Chinese Medicine (PZSHUTCM2212140006, Shanghai, China).

For 6‐OHDA induced PD models, the mice were divided into five groups: saline, 6‐OHDA, 6‐OHDA+PPX 1 mg kg^−1^/day, 6‐OHDA+PAE 50 mg kg^−1^/day, 6‐OHDA+PAE 100 mg kg^−1^/day (0.5% CMC‐Na was used as vehicle). Before the surgical procedure, 6‐OHDA+PPX and 6‐OHDA+PAE groups were orally preadministered with PPX or PAE for 3 d. 6‐OHDA was dissolved in saline solution at a concentration of 4 µg µL^−1^. The mice were anesthetized by inhalation of chloroflurane throughout the surgical procedure. Except for the saline group, each mouse was injected with 2.5 µL (1 µL min^−1^) of 6‐OHDA solution into the right SNpc according to the following coordinates (mm): anteroposterior (AP), −3.0; mediolateral (ML), ±1.3; dorsoventral (DV), −4.2/− 4.0/− 3.8 (all millimeters relative to bregma).^[^ ^51^ ^]^ Saline group was injected with equal volume of saline using same method. PPX and PAE were orally administrated to mice until completing all behavioral tests. The brain tissue was collected after behavioral tests were completed.

For MPTP (UHN, China) induced PD models, the mice were divided into four groups: saline, MPTP, MPTP +PPX 1 mg kg^−1^/day, MPTP +PAE 100 mg kg^−1^/day (0.5% CMC‐Na was used as vehicle). MPTP +PPX and MPTP +PAE groups were orally preadministered with PPX or PAE for 3 d. Except for the saline group, each mouse was injected intraperitoneally with MPTP (27.5 mg kg^−1^/day) for 5 d. Saline group was injected with equal volume of saline by the same method. PPX and PAE were orally administrated to mice until completing all behavioral tests after MPTP lesion.

### Behavioral Tests of Mice

Behavioral tests were performed as previously described.^[^ ^52^ ^]^ Briefly, for pole test, each mouse was placed on the small ball (diameter 2.5 cm) at the top of pole (height 52 cm diameter 1 cm). The total time to turn and descend were recorded. Each mouse was trained before the test and the test will be repeated twice.

For beam Test, each mouse was placed on the beginning of the beam (length 50 cm diameter 1 cm) 0.5 m above the ground. The total time to cross the beam was recorded. Each mouse was trained before the test and the test was repeated twice.

For rotarod test, each mouse was placed on a rotating rod that gradually accelerated from 0 to 40 rpm. The number of drops were recorded in 5 min. Each mouse was trained with rotation speeds of 5 rpm for 5 min.

### Immunofluorescence

Immunofluorescence was performed as previously described.^[^ ^35^ ^]^ Briefly, cells were fixed in 4% paraformaldehyde, blocked, and incubated with primary antibodies overnight at 4 °C. Then cells were washed with PBS and incubated with secondary antibody conjugated to Alexa Fluor 488 or 594 (Cell Signaling Technology, 4408S, Danvers, MA, USA). Cell nuclei were stained with DAPI (Yeasen, 40728ES03, Shanghai, China). Images were acquired with a Nikon spinning disk microscope with a 60×oil immersion objective. Fluorescence intensity was obtained using FIJI (National Institutes of Health, Bethesda, MD, USA).

Brain slices with a thickness of 35 µm prepared by frozen slicer. After washing with 0.1% Triton, the brain slices were blocked, then incubated with primary antibody against TH (1:200, Abcam) overnight at 4 °C. The fluorochrome‐conjugated secondary antibody Alexa Fluor 594 AffiniPure Goat Anti‐Rabbit IgG (1:500 Jackson immunoresearch, USA) was added and incubated for 90 min. The brain slices were sealed with antifade solution and visualized with a fluorescence microscope. For colocalization of TH with TIA‐1/p‐eIF2α, the primary antibody used anti‐TH (1:200, CST, USA) and anti‐TIA‐1 (1:300, ImmunoWay, USA) or anti‐p‐eIF2α (1:300, ImmunoWay, USA), and the fluorochrome‐conjugated secondary antibody used Alexa Fluor 594 AffiniPure Goat Anti‐Rabbit IgG (1:500 Jackson immunoresearch, USA) and Alexa Fluor 488 AffiniPure Goat Anti‐Mouse IgG (1:500 Jackson immunoresearch, USA), antifade solution added DAPI (Coolaber, SL1841, China).

### Statistical Analysis

All statistical analyses were done using OriginPro (2019b, OriginLab, Northampton, USA), GraphPad Prism 9.0 (GraphPad Software, Inc., La Jolla, CA), or Microsoft Excel (Professional 2019, Microsoft Corporation, Redmond, USA). All statistical values are displayed as the mean ± SEM, and the details are included in the corresponding figure legends. Volcano plots are drawn between untreated group versus 6‐OHDA treated group. Significantly changed proteins (differential hits) were called using a Student's *t*‐test with FDR *p*‐value < 0.05 and a minimum 1.5‐fold change. Differences between two groups were analyzed using a Student's *t*‐test. Multiple group comparisons were performed using a one‐way analysis of variance (ANOVA) followed by post‐hoc tests. Differences were considered significant at *p* < 0.05. P value significance is represented as follows: *p < 0.05, **p < 0.01, ***p < 0.001, n.s., no significance.

## Conflict of Interest

The authors declare no conflict of interest.

## Supporting information



Supporting Information

## Data Availability

The data that support the findings of this study are available from the corresponding author upon reasonable request.

## References

[1] a) B. R. Bloem , M. S. Okun , C. Klein , Lancet 2021, 397, 2284; 33848468 10.1016/S0140-6736(21)00218-X

[2] F. Wang , J. Li , S. Fan , Z. Jin , C. Huang , Pharmacol. Res. 2020, 161, 105143. 32814168 10.1016/j.phrs.2020.105143 PMC7428673

[3] a) H. Mahboubi , U. Stochaj , Biochim. Biophys. Acta, Mol. Basis Dis 2017, 1863, 884; 28095315 10.1016/j.bbadis.2016.12.022

[4] K. Thedieck , B. Holzwarth , M. T. Prentzell , C. Boehlke , K. Klasener , S. Ruf , A. G. Sonntag , L. Maerz , S. N. Grellscheid , E. Kremmer , R. Nitschke , E. W. Kuehn , J. W. Jonker , A. K. Groen , M. Reth , M. N. Hall , R. Baumeister , Cell 2013, 154, 859. 23953116 10.1016/j.cell.2013.07.031

[5] D. S. W. Protter , R. Parker , Trends Cell Biol. 2016, 26, 668. 27289443 10.1016/j.tcb.2016.05.004 PMC4993645

[6] a) Q. Cui , H. Bi , Z. Lv , Q. Wu , J. Hua , B. Gu , C. Huo , M. Tang , Y. Chen , C. Chen , S. Chen , X. Zhang , Z. Wu , Z. Lao , N. Sheng , C. Shen , Y. Zhang , Z. Y. Wu , Z. Jin , P. Yang , H. Liu , J. Li , G. Bai , Cell 2023, 186, 803; 36738734 10.1016/j.cell.2022.12.046

[7] a) B. Wolozin , P. Ivanov , Nat. Rev. Neurosci. 2019, 20, 649; 31582840 10.1038/s41583-019-0222-5 PMC6986315

[8] a) S. Hofmann , N. Kedersha , P. Anderson , P. Ivanov , Biochim. Biophys. Acta, Mol. Cell Res. 2021, 1868, 118876; 33007331 10.1016/j.bbamcr.2020.118876 PMC7769147

[9] a) P. Yang , C. Mathieu , R. M. Kolaitis , P. Zhang , J. Messing , U. Yurtsever , Z. Yang , J. Wu , Y. Li , Q. Pan , J. Yu , E. W. Martin , T. Mittag , H. J. Kim , J. P. Taylor , Cell 2020, 181, 325; 32302571 10.1016/j.cell.2020.03.046 PMC7448383

[10] S. Markmiller , S. Soltanieh , K. L. Server , R. Mak , W. Jin , M. Y. Fang , E. C. Luo , F. Krach , D. Yang , A. Sen , A. Fulzele , J. M. Wozniak , D. J. Gonzalez , M. W. Kankel , F. B. Gao , E. J. Bennett , E. Lecuyer , G. W. Yeo , Cell 2018, 172, 590. 29373831 10.1016/j.cell.2017.12.032 PMC5969999

[11] a) J. Gal , L. Kuang , K. R. Barnett , B. Z. Zhu , S. C. Shissler , K. V. Korotkov , L. J. Hayward , E. J. Kasarskis , H. Zhu , Acta Neuropathol. 2016, 132, 563; 27481264 10.1007/s00401-016-1601-x PMC5023729

[12] a) R. Koppenol , A. Conceicao , I. T. Afonso , R. Afonso‐Reis , R. G. Costa , S. Tome , D. Teixeira , J. P. da Silva , J. M. Codesso , D. V. C. Brito , L. Mendonca , A. Marcelo , L. Pereira de Almeida , C. A. Matos , C. Nobrega , Brain 2023, 146, 2346; 36511898 10.1093/brain/awac473 PMC10232246

[13] S. Martin , L. Zekri , A. Metz , T. Maurice , K. Chebli , M. Vignes , J. Tazi , J. Neurochem. 2013, 125, 175. 23373770 10.1111/jnc.12189

[14] S. Anisimov , M. Takahashi , T. Kakihana , Y. Katsuragi , H. Kitaura , L. Zhang , A. Kakita , M. Fujii , Sci. Rep.‐Uk 2019, 9, 12896. 10.1038/s41598-019-46237-1 PMC6733845 31501480

[15] S. Kwon , Y. Zhang , P. Matthias , Genes Dev. 2007, 21, 3381. 18079183 10.1101/gad.461107 PMC2113037

[16] M. Saito , D. Hess , J. Eglinger , A. W. Fritsch , M. Kreysing , B. T. Weinert , C. Choudhary , P. Matthias , Nat. Chem. Biol. 2019, 15, 51. 30531905 10.1038/s41589-018-0180-7

[17] J. Gal , J. Chen , D. Y. Na , L. Tichacek , K. R. Barnett , H. Zhu , Mol. Cell. Biol. 2019, 39, e00052‐19. 31481451 10.1128/MCB.00052-19 PMC6817755

[18] S. Mazzetti , M. De Leonardis , G. Gagliardi , A. M. Calogero , M. J. Basellini , L. Madaschi , I. Costa , F. Cacciatore , S. Spinello , M. Bramerio , R. Cilia , C. Rolando , G. Giaccone , G. Pezzoli , G. Cappelletti , Front. Neurosci. 2020, 14, 624. 32655357 10.3389/fnins.2020.00624 PMC7324673

[19] a) V. K. Godena , N. Brookes‐Hocking , A. Moller , G. Shaw , M. Oswald , R. M. Sancho , C. C. Miller , A. J. Whitworth , K. J. De Vos , Nat. Commun. 2014, 5, 5245; 25316291 10.1038/ncomms6245 PMC4208097

[20] a) Y. Fuyuno , H. Uchi , M. Yasumatsu , S. Morino‐Koga , Y. Tanaka , C. Mitoma , M. Furue , Oxid. Med. Cell. Longevity 2018, 2018, 6091947; 10.1155/2018/6091947 PMC5987232 29951165

[21] J. X. Zhu , W. Q. Hu , S. Q. Dong , L. T. Yi , J. X. Zeng , M. Li , Pharmacol. Rep. 2019, 71, 430. 31003153 10.1016/j.pharep.2019.01.009

[22] Y. Qiu , X. J. Xue , G. Liu , M. M. Shen , C. Y. Chao , J. Zhang , Y. Q. Guo , Q. Q. Niu , Y. N. Yu , Y. T. Song , H. H. Wang , S. X. Wang , Y. J. Chen , L. H. Jiang , P. Li , Y. L. Yin , Chin. Med. 2021, 16, 136. 34903262 10.1186/s13020-021-00545-9 PMC8670250

[23] L. X. Xu , Y. B. Li , Q. Fu , S. P. Ma , Biochem. Biophys. Res. Commun. 2014, 454, 65. 25445600 10.1016/j.bbrc.2014.10.025

[24] W. J. Zheng , B. Liu , E. Y. Shi , J. Surg. Res. 2021, 268, 308. 34399353 10.1016/j.jss.2021.06.055

[25] N. Gupta , M. Badeaux , Y. Liu , K. Naxerova , D. Sgroi , L. L. Munn , R. K. Jain , I. Garkavtsev , Proc. Natl. Acad. Sci. 2017, 114, 1033. 28096337 10.1073/pnas.1525387114 PMC5293063

[26] a) G. A. Corbet , J. M. Burke , G. R. Bublitz , J. W. Tay , R. Parker , Proc. Natl. Acad. Sci. USA 2022, 119, e2204235119; 35939694 10.1073/pnas.2204235119 PMC9388085

[27] a) X. Yang , Z. Hu , S. Fan , Q. Zhang , Y. Zhong , D. Guo , Y. Qin , M. Chen , PLoS Pathog. 2018, 14, e1006901; 29415027 10.1371/journal.ppat.1006901 PMC5819834

[28] J. R. Wheeler , T. Matheny , S. Jain , R. Abrisch , R. Parker , Elife 2016, 5, e18413. 27602576 10.7554/eLife.18413 PMC5014549

[29] C. T. Kuo , G. T. You , Y. J. Jian , T. S. Chen , Y. C. Siao , A. L. Hsu , T. T. Ching , Aging Cell 2020, 19, e13157. 32432401 10.1111/acel.13157 PMC7294782

[30] a) K. Arimoto , H. Fukuda , S. Imajoh‐Ohmi , H. Saito , M. Takekawa , Nat. Cell Biol. 2008, 10, 1324; 18836437 10.1038/ncb1791

[31] a) M. Jedrusik‐Bode , M. Studencka , C. Smolka , T. Baumann , H. Schmidt , J. Kampf , F. Paap , S. Martin , J. Tazi , K. M. Muller , M. Kruger , T. Braun , E. Bober , J. Cell Sci. 2013, 126, 5166; 24013546 10.1242/jcs.130708

[32] a) A. C. Goncalves , E. R. Towers , N. Haq , J. A. Porco Jr. , J. Pelletier , S. J. Dawson , J. E. Gale , Sci. Rep. 2019, 9, 12501; 31467369 10.1038/s41598-019-48393-w PMC6715625

[33] a) T. A. Shelkovnikova , P. Dimasi , M. S. Kukharsky , H. An , A. Quintiero , C. Schirmer , L. Buee , M. C. Galas , V. L. Buchman , Cell Death Dis. 2017, 8, e2788; 28492545 10.1038/cddis.2017.199 PMC5520719

[34] a) P. Bhadauriya , R. Parihar , S. Ganesh , Biochem. Biophys. Rep. 2021, 28, 101110; 34485711 10.1016/j.bbrep.2021.101110 PMC8405967

[35] L. Luo , Z. Li , T. Zhao , X. Ju , P. Ma , B. Jin , Y. Zhou , S. He , J. Huang , X. Xu , Y. Zou , P. Li , A. Liang , J. Liu , T. Chi , X. Huang , Q. Ding , Z. Jin , C. Huang , Y. Zhang , Sci. Bull. (Beijing) 2021, 66, 1194. 33495715 10.1016/j.scib.2021.01.013 PMC7816596

[36] S. Anisimov , M. Takahashi , T. Kakihana , Y. Katsuragi , H. Kitaura , L. Zhang , A. Kakita , M. Fujii , Sci. Rep. 2019, 9, 12896. 31501480 10.1038/s41598-019-46237-1 PMC6733845

[37] a) S. Ray , N. Singh , R. Kumar , K. Patel , S. Pandey , D. Datta , J. Mahato , R. Panigrahi , A. Navalkar , S. Mehra , L. Gadhe , D. Chatterjee , A. S. Sawner , S. Maiti , S. Bhatia , J. A. Gerez , A. Chowdhury , A. Kumar , R. Padinhateeri , R. Riek , G. Krishnamoorthy , S. K. Maji , Nat. Chem. 2020, 12, 705; 32514159 10.1038/s41557-020-0465-9

[38] H. Sidibe , A. Dubinski , C. Vande Velde , J. Neurochem. 2021, 157, 944. 33349931 10.1111/jnc.15280 PMC8248322

[39] M. Lemos , N. Stefanova , Front. Synaptic Neurosci. 2020, 12, 586453. 33041780 10.3389/fnsyn.2020.586453 PMC7518386

[40] J. Zhao , Y. He , Y. Duan , Y. Ma , H. Dong , X. Zhang , R. Fang , Y. Zhang , M. Yu , F. Huang , Int. J. Mol. Sci. 2023, 24. 10.3390/ijms24129975 PMC10298689 37373121

[41] S. Yan , X. Wei , W. Jian , Y. Qin , J. Liu , S. Zhu , F. Jiang , H. Lou , B. Zhang , Front. Aging Neurosci. 2020, 12, 78. 32296327 10.3389/fnagi.2020.00078 PMC7137996

[42] Z. Li , L. Luo , X. Ju , S. Huang , L. Lei , Y. Yu , J. Liu , P. Zhang , T. Chi , P. Ma , C. Huang , X. Huang , Q. Ding , Y. Zhang , EMBO J. 2024, 43, 6444. 39567830 10.1038/s44318-024-00314-y PMC11649915

[43] J. Eberhardt , D. Santos‐Martins , A. F. Tillack , S. Forli , J. Chem. Inf. Model. 2021, 61, 3891. 34278794 10.1021/acs.jcim.1c00203 PMC10683950

[44] P. A. Ravindranath , S. Forli , D. S. Goodsell , A. J. Olson , M. F. Sanner , PLoS Comput. Biol. 2015, 11, e1004586. 26629955 10.1371/journal.pcbi.1004586 PMC4667975

[45] D. Seeliger , B. L. de Groot , J. Comput.‐Aided Mol. Des. 2010, 24, 417. 20401516 10.1007/s10822-010-9352-6 PMC2881210

[46] S. He , H. Gou , Y. Zhou , C. Wu , X. Ren , X. Wu , G. Guan , B. Jin , J. Huang , Z. Jin , T. Zhao , FASEB J. 2023, 37, e23269. 37889852 10.1096/fj.202201973RR

[47] T. Stiernagle , WormBook 2006, 1. 10.1895/wormbook.1.101.1 PMC4781397 18050451

[48] Y. Liu , Z. Zhou , L. Yin , M. Zhu , F. Wang , L. Zhang , H. Wang , Z. Zhou , H. Zhu , C. Huang , S. Fan , BioFactors 2022, 48, 442. 34580918 10.1002/biof.1788

[49] J. F. Cooper , J. M. Van Raamsdonk , J. Parkinsons Dis. 2018, 8, 17. 29480229 10.3233/JPD-171258 PMC5836411

[50] H. Li , Y. Feng , Z. Chen , X. Jiang , Z. Zhou , J. Yuan , F. Li , Y. Zhang , X. Huang , S. Fan , X. Wu , C. Huang , Pharmacol. Res. 2021, 163, 105220. 33007422 10.1016/j.phrs.2020.105220

[51] a) J. Zhu , Z. Hu , X. Han , D. Wang , Q. Jiang , J. Ding , M. Xiao , C. Wang , M. Lu , G. Hu , Cell Death Differ. 2018, 25, 2037; 29786071 10.1038/s41418-018-0127-2 PMC6219479

[52] X. L. Wang , S. T. Feng , Y. T. Wang , N. N. Zhang , Z. Y. Guo , X. Yan , Y. H. Yuan , Z. Z. Wang , N. H. Chen , Y. Zhang , Phytomedicine 2022, 104, 154281. 35752080 10.1016/j.phymed.2022.154281

