# Supplementary material for: Perillaldehyde Improves Parkinson‐Like Deficits by Targeting G3BP Mediated Stress Granule Assembly in Preclinical Models
Source: Adv Sci (Weinh). 2025 Feb 14;12(14):2412152. doi: 10.1002/advs.202412152 (PMC11984871; doi:10.1002/advs.202412152)
Supplement: Supplementary file 1 — Supporting Information [file ADVS-12-2412152-s001.pdf]

ADVANCED SCIENCE Open Access

Supporting Information

for *Adv. Sci.*, DOI 10.1002/advs.202412152

Perillaldehyde Improves Parkinson-Like Deficits by Targeting G3BP Mediated Stress Granule Assembly in Preclinical Models

Minglv Fang, Lingling Luo, Youjia Chen, Ying Liu, Yingxuan Yan, Fei Wang, Yan Zou, Huanhu Zhu, Xiaojun Wu, Zhigang Jin, Cheng Huang*, Yu Zhang* and Shengjie Fan**

Supplementary Information for

Perillaldehyde Improves Parkinson-like Deficits by Targeting G3BP Mediated Stress Granule Assembly in Preclinical Models

Minglv Fang[1#], Lingling Luo[1,2,3#], Youjia Chen[4#], Ying Liu[1], Yingxuan Yan[1], Fei Wang[1], Yan Zou[2], Huanhu Zhu[2], Xiaojun Wu[1], Zhigang Jin[4*], Cheng Huang[1*], Yu Zhang[5*], Shengjie Fan[1*]

1 School of Pharmacy, Shanghai University of Traditional Chinese Medicine, Shanghai 201203, China.

2 School of Life Science and Technology, ShanghaiTech University, Shanghai 201210, China.

3 The Affiliated Hospital of Jiangxi University of Traditional Chinese Medicine, Nanchang 330006, China.

4 College of Life Sciences, Zhejiang Normal University, Jinhua 321004, China.

5 Shanghai-MOST Key Laboratory of Health and Disease Genomics, NHC Key Lab of Reproduction Regulation, Shanghai Institute for Biomedical and Pharmaceutical Technologies, Shanghai 200237, China.

# These authors contributed equally

*Correspondence: zgkin@zjnu.edu.cn; chuang@shutcm.edu.cn; zhangy@shanghaitech.edu.cn; shengjiefan@shutcm.edu.cn

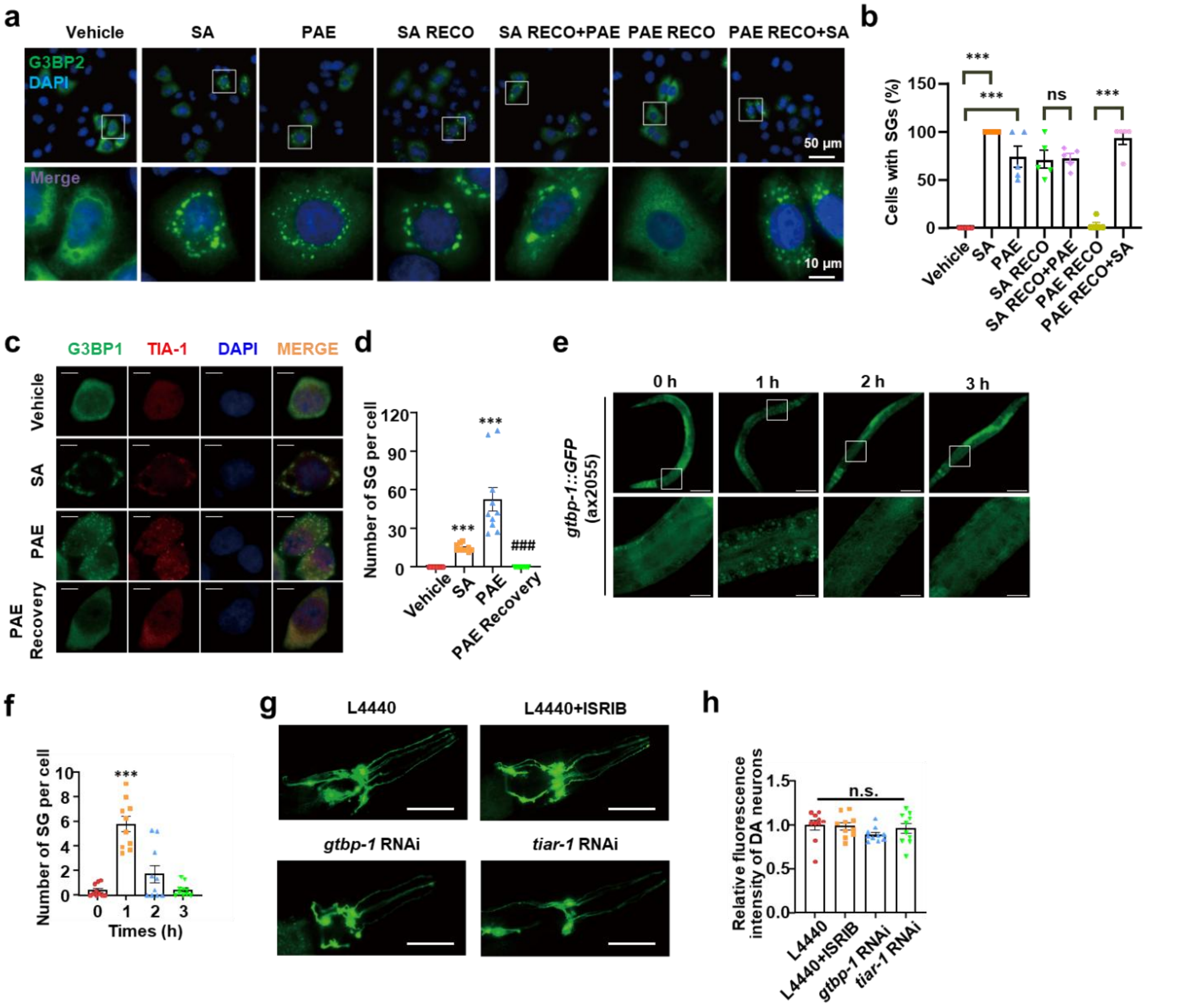

Figure S1.

PAE-induced SG dynamics *in vitro* and *in vivo*. a) The dynamics of SG recovery in PAE-treated cultures. EGFP-G3BP2/HeLa cells were treated with SA or PAE for 1 hour, then the cells were allowed to recover for 1 hour. After that, these stressed cells were treated with SA or PAE for additional 1 hour. b) Quantification of the experiment performed in panel (a). Each dot represents percent of SG-positive cells, n = 5. c) SH-SY5Y cells were treated with SA or PAE for 1 hour, then the cells treated with PAE allowed to recover for 1 hour. Colocalization study of SG maker: G3BP1 and TIA-1 via immunofluorescence in SH-SY5Y with the above conditions. Scale bars: 10 μm. d) Quantification of the experiment performed in panel (c). Each dot represents number of SGs per cell, n = 10. e) Transgenic worms *gtbp-1::GFP* were cultivated until L3 stage, and SG formation was captured after the worms were treated with PAE for 0, 1, 2, 3 h. Scale bars: 50 μm, 10 μm (enlarged image). f) Quantification of the experiment performed in panel (e). Each dot represents average number of SGs in 10~15 cells /worm, n = 10. g) Transgenic worms UA57 were treated or untreated with ISRIB, *gtbp-1* RNAi and *tiar-1* RNAi. h) Quantification of the experiment performed

in panel (g). Each dot represents relative fluorescence intensity of dopaminergic neurons (fold of vehicle) measured by ImageJ software, n = 10. Data are shown as the mean ± SEM, *$p<0.05$ *vs* vehicle, ***$p<0.001$ *vs* vehicle, ###$p<0.001$ *vs* PAE, n.s., not significant.

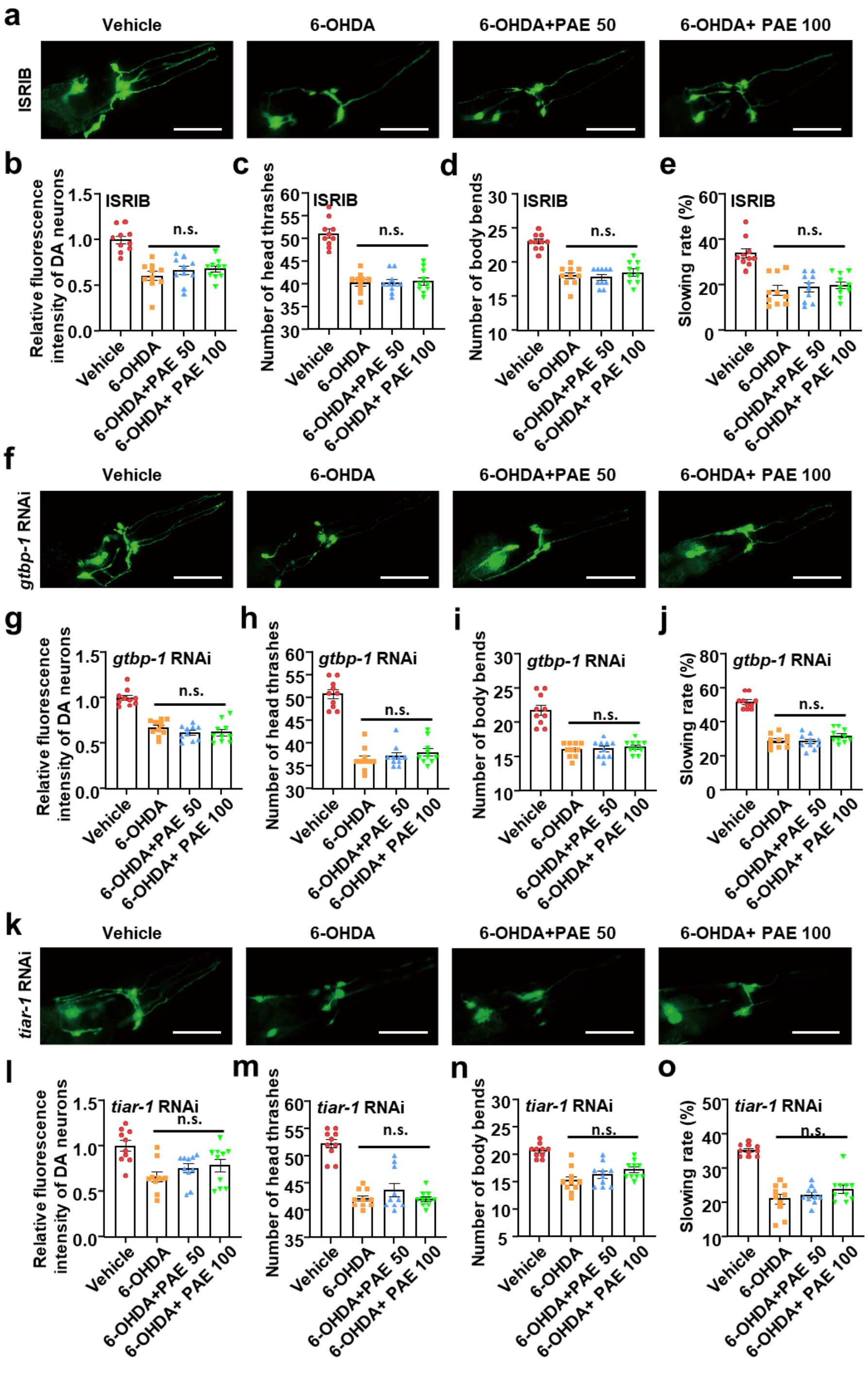

Figure S2.

SGs are indispensable for the neuroprotective effect of PAE on 6-OHDA-induced *C. elegans* models of PD. a) Transgenic worms UA57 were treated with PAE combined with ISRIB and then exposed to 6-OHDA. b) Quantification of the experiment performed in panel (a). Column charts display relative fluorescence intensity of dopaminergic neurons (fold of vehicle) measured by ImageJ

software, n = 10. c-e**).** Behavioral phenotyping of worms was observed after 72 h treatment of 6-OHDA and ISRIB. Column charts respectively display, from left to right, number of head thrashes / 20 s in M9 buffer, number of body bends / 20 s in a NGM plate without food, slowing rate / 20 s, n = 10. f) Transgenic worms UA57 were fed with *gtbp-1* RNAi bacteria mixed with PAE and then exposed to 6-OHDA. g) Quantification of the experiment performed in panel (f). Column charts display relative fluorescence intensity of dopaminergic neurons (fold of vehicle) measured by ImageJ software, n = 10. h-j) Behavioral phenotyping of worms was observed after 72 h treatment of 6-OHDA and *gtbp-1* RNAi. Column charts respectively display, from left to right, number of head thrashes / 20 s in M9 buffer, number of body bends / 20 s in a NGM plate without food, slowing rate / 20 s, n = 10. k) Transgenic worms UA57 were fed with *tiar-1* RNAi bacteria mixed with PAE and then exposed to 6-OHDA. l) Quantification of the experiment performed in panel (k). Column charts display relative fluorescence intensity of dopaminergic neurons (fold of vehicle) measured by ImageJ software, n = 10. m-o) Behavioral phenotyping of worms was observed after 72 h treatment of 6-OHDA and *tiar-1* RNAi. Column charts respectively display, from left to right, number of head thrashes / 20 s in M9 buffer, number of body bends / 20 s in a NGM plate without food, slowing rate / 20 s, n = 10. Data are shown as the mean ± SEM, n.s., not significant.

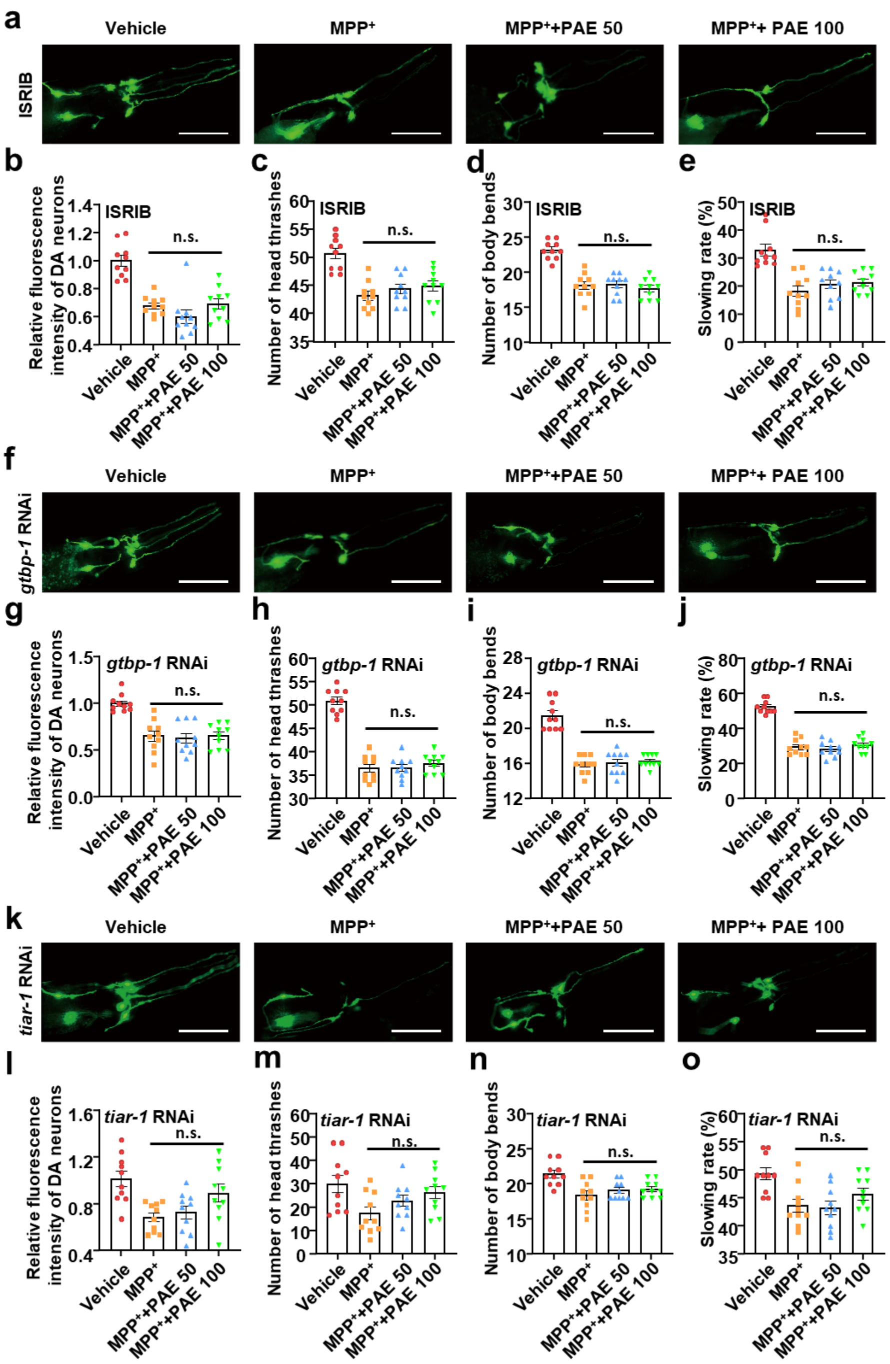

Figure S3.

SGs are indispensable for the neuroprotective effect of PAE on MPP^+-induced *C. elegans* models of PD. a) Transgenic worm UA57 were treated with PAE combined with ISRIB and then exposed to MPP^+. b) Quantification of the experiment performed in panel (a). Column charts display relative fluorescence intensity of dopaminergic neurons (fold of vehicle) measured by ImageJ software, n = 10. c-e) Behavioral phenotyping of worms was observed after 72 h treatment of MPP^+ and ISRIB.

Column charts respectively display, from left to right, number of head thrashes / 20 s in M9 buffer, number of body bends / 20 s in a NGM plate without food, slowing rate / 20 s, n = 10. f-g) Fluorescence intensity of dopaminergic neurons in UA57 when fed with *gtbp-1* RNAi bacteria mixed with PAE and then exposed to MPP^+, n = 10. h-j) Behavioral phenotyping of worms was observed after 72 h treatment of MPP^+ and *gtbp-1* RNAi. Column charts respectively display, from left to right, number of head thrashes, body bends and slowing rate, n = 10. k-l) Dopaminergic neuron intensity in *tiar-1* RNAi fed UA57 with PAE and then exposed to MPP^+, n = 10. m-o) Behavioral phenotyping of worms was observed after 72 h treatment of MPP^+ and *tiar-1* RNAi. Column charts respectively display, from left to right, number of head thrashes, number of body bends, slowing rate , n = 10. Data are shown as the mean ± SEM, n.s., not significant.

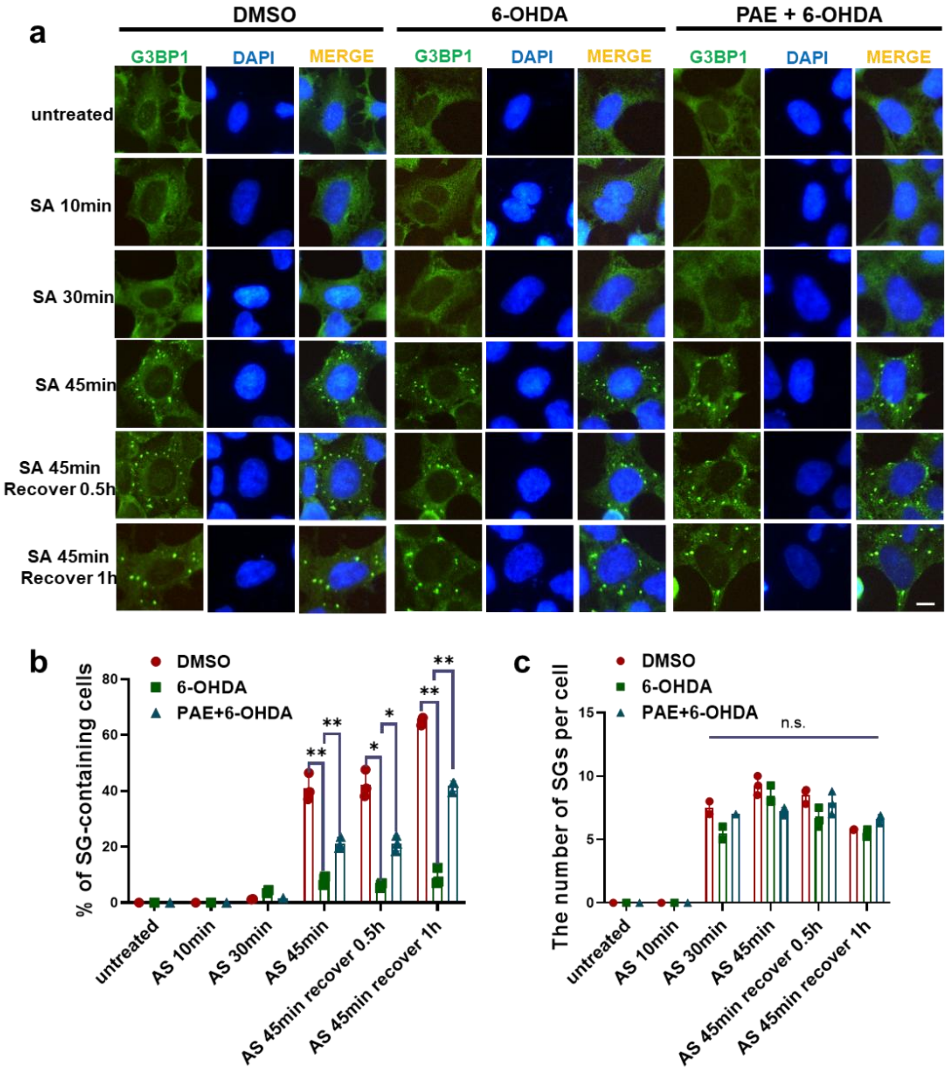

Figure S4.
PAE rescued 6-OHDA-induced defect in SG assembly. a) SH-SY5Y cells were pre-treated with DMSO or 100 μM PAE for 1 h, then treated with DMSO or 100 μM 6-OHDA for 24 h and followed by SG induction with 0.5 mM SA and SG recovery by removal of SA. Cells at different SG stages were harvested for immunostaining of G3BP1 (green). Scale bars: 10 μm. b,c) Statistical analysis of the percentage of SG-containing cells and the number of SG per SG-containing cell as shown in panel (a), n = 3. Data are shown as the mean ± SEM, *$p < 0.05$, **$p < 0.01$, n.s., not significant.

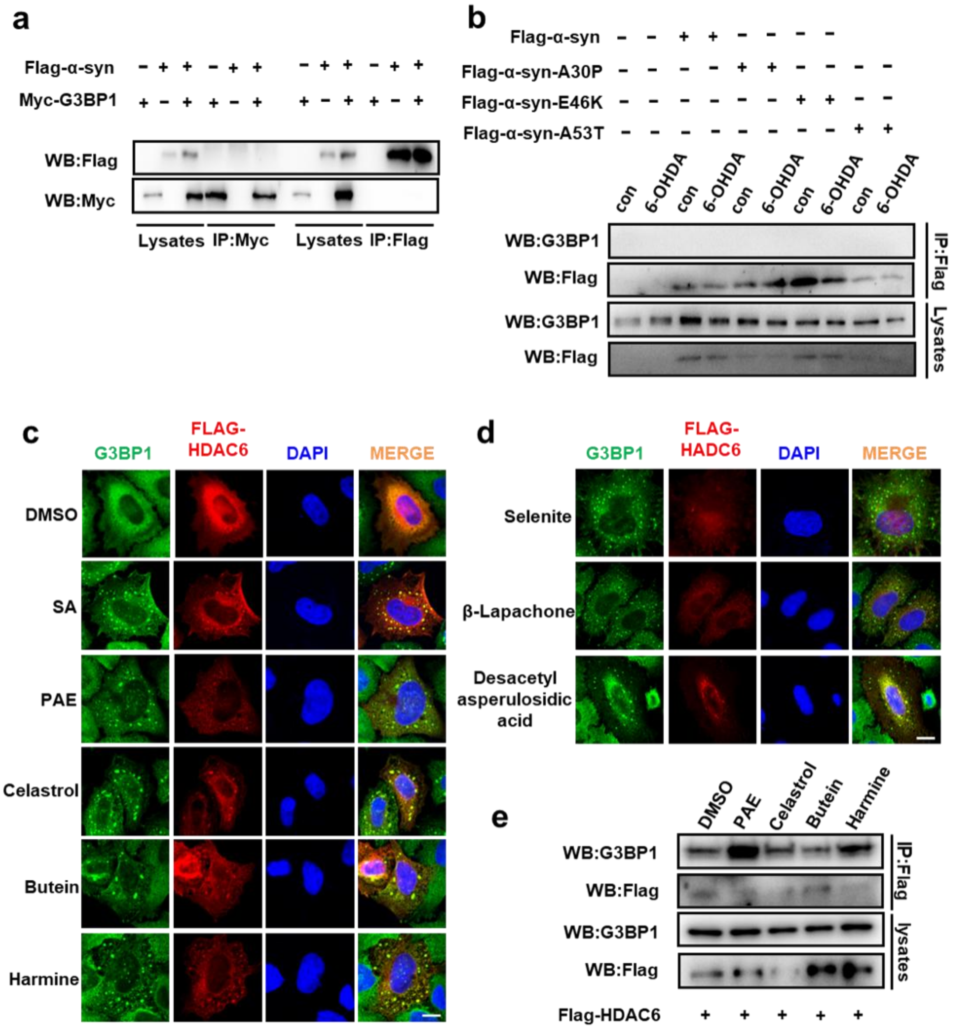

Figure S5.

PAE recruits HDAC6 to SGs. a) HEK293T cells transfected with pCS2-Flag-α-synuclein, pCS2-Myc-G3BP1 or both were subjected to immunoprecipitation with an anti-Myc or anti-Flag antibody. The presence of α-syn and G3BP1 in the immunoprecipitates was assessed by western blotting with anti-Flag and anti-Myc antibodies, respectively. b) SH-SY5Y cells transfected with expression vectors of wild-type or mutant α-syn were treated with DMSO or 100 μM 6-OHDA for 24 h, and subjected to immunoprecipitation with anti-Flag antibody. c,d) A subset of SG inducers recruit HDAC6 to SGs. HeLa cells transfected with pCS2-Flag-HDAC6 were treated with 0.5 mM SA for 45 min, 100 μM PAE for 1 h, 1 mM selenite for 2 h, 10 μM Celastrol, Butein, Harmine, β-Lapachone or Desacetyl asperulosidic acid for 2 h followed by immunostaining for G3BP1 (green) and Flag (red). Scale bars: 10 μm. e) After transfection with pCS2-Flag-HDAC6 for 24 h, cells were pre-treated with DMSO, 100 μM PAE for 1 h, 10 μM celastrol, butein or harmine for 2 h and incubated in normal growth medium for additional 24 h and subjected to immunoprecipitation with

an anti-Flag antibody. The presence of G3BP1 in the immunoprecipitates was assessed by western blotting with an anti-G3BP1 antibody.

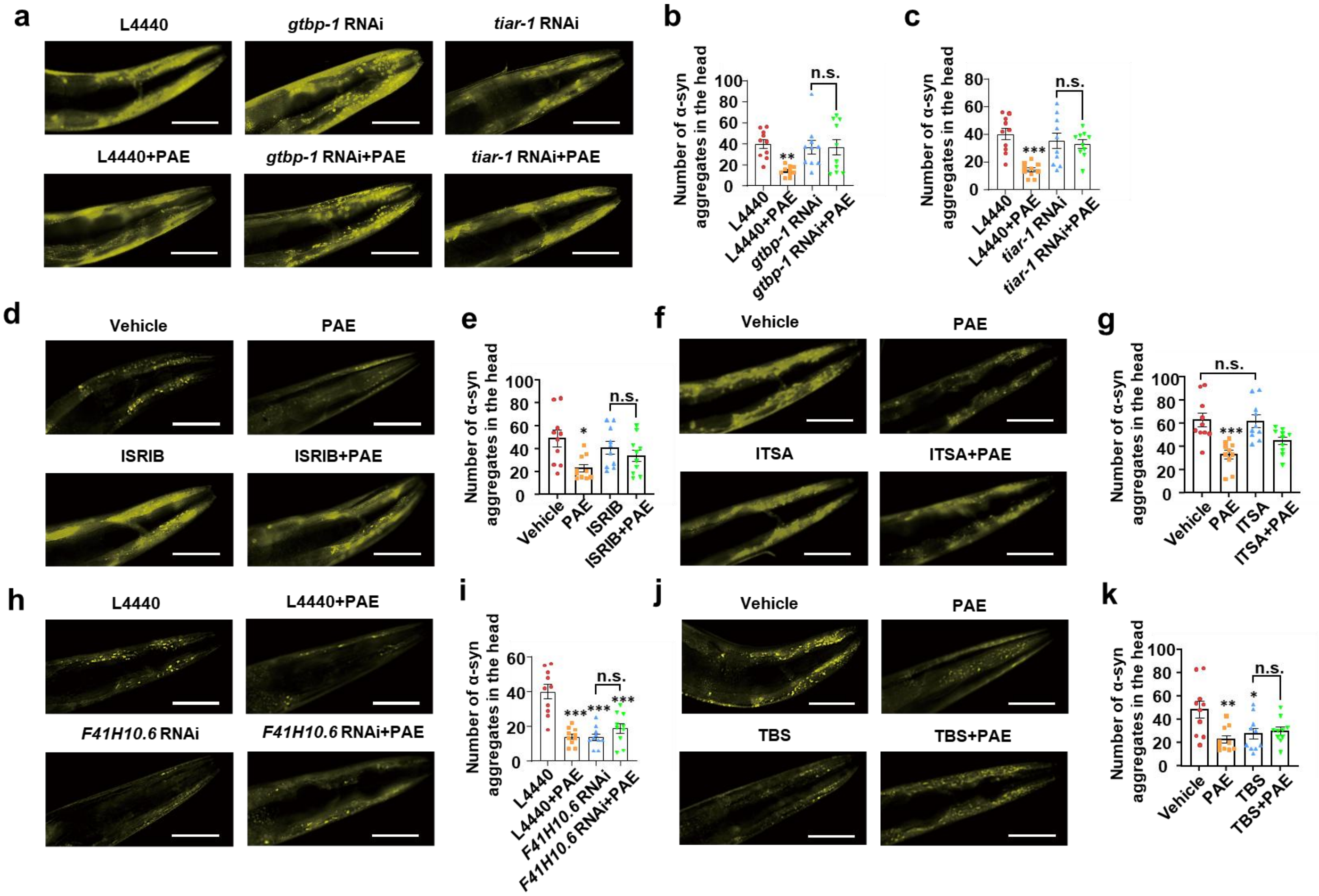

Figure S6.

The effect of SG inhibition and HDAC6 on α-syn aggregation in worms. a) Transgenic worm NL5901 were fed with L4440 or *gtbp-1* or *tiar-1* RNAi bacteria mixed with or without PAE. b-c) Quantification of the experiment performed in panel (a). Column charts display the number of α-syn aggregates in the head of worms, n = 10. d-e). α-syn aggregation in NL5901 when treated with ISRIB and/or PAE from L1 to Day 5. f-g**).** α-syn aggregation in NL5901 when treated ITSA-1 and/or PAE from L1 to Day 5. h-i) α-syn aggregation in NL5901 when fed L4440, *F41H10.6* (*hda-6*) RNAi bacteria mixed with or without PAE. j-k) α-syn aggregation in NL5901 when treated with TBS and PAE from L1 to Day 5. All data are shown as the mean ± SEM, n=10, ***p<0.001 *vs* vehicle, **p<0.01 *vs* vehicle, *p<0.05 *vs* vehicle, n.s., not significant. Scale bars: 100 μm.

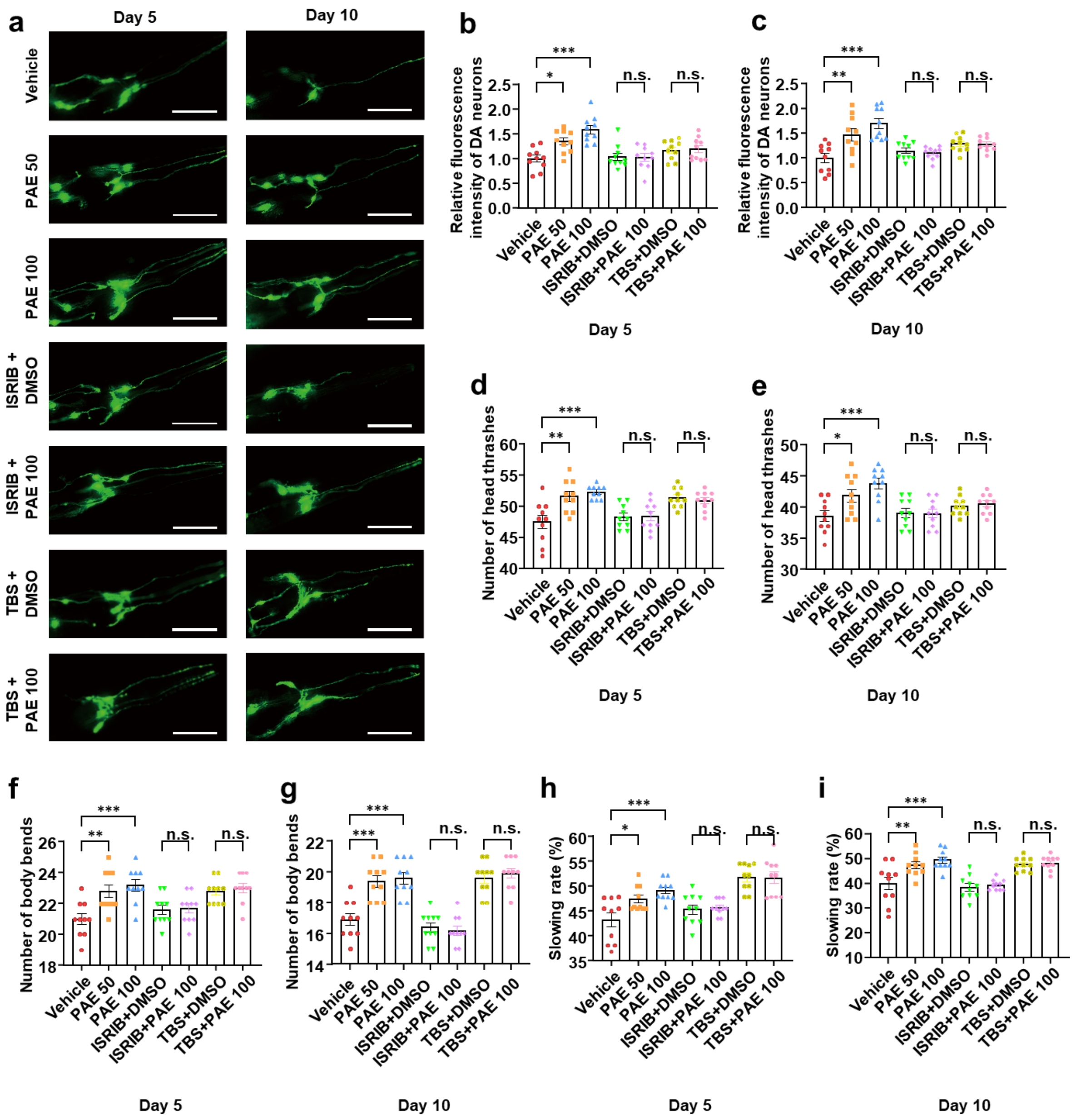

Figure S7.

Deficiency of *hda-6* attenuates the neuroprotective effect of PAE in α-syn aggregation *C. elegans*. a) Dopaminergic neurons (GFP-labeled) in transgenic UA44 worms when treated with ISRIB, TBS and PAE until adult Day 5 and Day 10. b-c) Quantification of the experiment performed in panel (a). Column charts display relative fluorescence intensity of dopaminergic neurons (fold of vehicle) measured by ImageJ software. d-e) Number of head thrashes / 20 s in M9 buffer. f-g) Number of body bends / 20 s in a NGM plate without food. h-i) Slowing rate / 20 s. All data are shown as the mean ± SEM, n=10, ****p*<0.001 *vs* vehicle, ***p*<0.01 *vs* vehicle, **p*<0.05 *vs* vehicle, n.s., not significant. Scale bars: 100 μm.

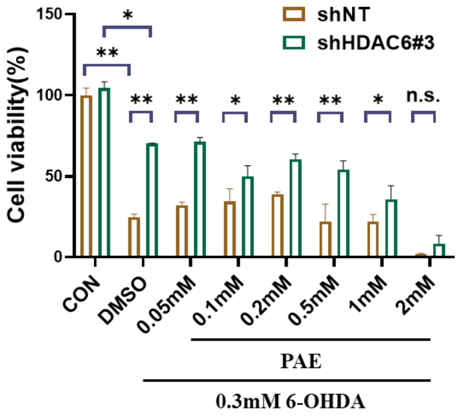

Figure S8.

PAE protects SH-SY6Y cells from 6-OHDA-induced neurotoxicity via inhibition of HDAC6. SH-SY5Y cells infected with HDAC6 shRNA (shHDAC6) or non-targeting control shRNA (shNT) were pretreated with different doses of PAE (0.05, 0.1, 0.2, 0.5, 1 and 2 mM) for 1h and exposed to 300 μM 6-OHDA for 24 h. Cell viability was assessed by CCK-8 assay. Data are shown as the mean ± SEM, n=3, ** $p<0.01$ *vs* vehicle, * $p<0.05$ *vs* vehicle, n.s., not significant.
